# Qinggan Yipi capsule ameliorates hepatic fibrosis in rats by down-regulating the TGF-β1/Smad2/3 signaling pathway and improving gut microbiota imbalance

**DOI:** 10.3389/fphar.2025.1525914

**Published:** 2025-01-24

**Authors:** Wenjing Xue, Haiqing Liu, Ziheng Su, Siqi Wang, Junping Cheng, Yunzhi Pan, Lurong Zhang

**Affiliations:** ^1^ Key Laboratory for Evaluation and Transformation of Wu Men Medical School’s Empirical Prescriptions, Suzhou Traditional Chinese Medicine Hospital, Affiliated to Nanjing University of Chinese Medicine, Suzhou, China; ^2^ Department of Pharmacy, Affiliated Hospital of Hebei University, Baoding, China; ^3^ Department of Pharmacy, Suzhou Fifth People’s Hospital, Suzhou, China

**Keywords:** Qinggan Yipi capsule, hepatic fibrosis, TGF-β1/Smad2/3 signaling pathway, gut microbiota, network pharmacology, HSC-T6

## Abstract

**Background and objective:**

Qinggan Yipi Capsule (QgYp) is a hospital preparation that has been used for many years in the treatment of chronic liver diseases. However, the mechanism of QgYp in ameliorating hepatic fibrosis (HF) remains unclear. This study aims to clarify the anti-liver fibrosis effect of QgYp and its mechanism of action.

**Methods:**

This study uses a carbon tetrachloride (CCl_4_) induced HF rat model and TGF-β1 stimulated HSC-T6 cell line (rat HSCs) as experimental models. The therapeutic effects were evaluated through pathology, biochemical tests, and ELISA. The therapeutic mechanism of QgYp for HF was predicted through network pharmacology. The expression of TGF-β1/Smad2/3 related proteins was detected by qPCR analysis and Western blot analysis. The composition of the gut microbiota was analyzed using 16S rRNA gene sequencing.

**Results:**

Histopathological analysis, serum biochemical tests, and ELISA measurements showed that QgYp effectively decreased the levels of ALT, AST, HA, LN, PCIII, and IV-C while improving collagen deposition and hepatocyte necrosis. Protein-protein interaction (PPI) network analysis screened HF-related genes, including peroxisome proliferator-activated receptor gamma (PPARG), tumor necrosis factor (TNF), and TGF-β1. GO and KEGG analyses indicated that QgYp significantly affects TGF-β signaling pathway. In addition, the results of qPCR and Western blot analysis from both *in vitro* and *in vivo* experiments indicated that QgYp significantly downregulated the expression of proteins and mRNA associated with the TGF-β1/Smad2/3 pathway. The 16S rDNA gene sequencing results showed that QgYp can increase the diversity and richness of the gut microbiota in HF rats and alter the composition of the gut microbiota.

**Conclusion:**

QgYp could effectively ameliorate HF, and this effect might be connected to the downregulation of the TGF-β1/Smad2/3 pathway, the suppression of HSCs activation, and regulation of gut microbiota dysbiosis.

## 1 Introduction

Hepatic fibrosis (HF) is a compensatory response to liver tissue repair and damage brought on by numerous chronic pathogenic causes (e.g., metabolic liver disease, chronic alcohol consumption, and viral hepatitis). It is a crucial step in the development of cirrhosis and hepatocellular carcinoma, leading to over 1 million deaths annually (Karmacharya et al., 2020; Roehlen et al., 2020; Zhao et al., 2022; Zhang et al., 2020). With the advancement of modern society, HF has gradually become one of the serious threats to human health.

Hepatic stellate cell (HSCs) activation is the primary factor in the development of HF. Quiescent HSCs become activated and transform into myofibroblasts in response to external stimuli. This transformation leads to an accumulation of extracellular matrix (ECM) and accelerates the development of HF. Research indicates that transforming growth factor-beta1 (TGF-β1) plays a crucial role in activating HSCs, which is a key factor in the development of HF (Zhao et al., 2022). TGF-β1 promotes the growth of HF by increasing Smad2 and Smad3 phosphorylation and activation, and by controlling the transcription of particular target genes (such as collagen-I) in the cell nucleus (Zhang et al., 2020; Liu F. et al., 2021). Therefore, regulating the TGF-β1/Smad2/3 signaling pathway and HSCs activation are considered effective strategies for treating HF currently (Zhang et al., 2022).

In addition, gut microbiota plays a crucial role in liver health (Abe et al., 2020). From an anatomical perspective, the liver is connected to the intestine through the portal vein and mesenteric lymphatic system. When the intestinal barrier is damaged, inflammatory mediators and microbial metabolites can enter the liver through the portal vein, triggering a series of immune reactions. Liver injury-induced abnormalities in bile acid metabolism and inflammatory responses may lead to impaired normal function of the intestinal barrier and dysbiosis of the gut microbiome (Bragazzi et al., 2023; Huang et al., 2024). Recent research indicates that an imbalance of gut microbiome significantly correlates with the severity of HF. Upon correction of its dysregulation, it can lead to the downregulation of the TGF-β/Smad signaling pathway in the liver, thereby inhibiting the activation of HSC (Zhang et al., 2017; Li et al., 2020; Wan et al., 2020). This suggests that gut microbiome alteration is also an important mechanism affecting HF (Liu et al., 2020; Depommier et al., 2019).

Currently, there are several drugs available for the treatment of HF. Existing chemical and biological agents, however, have limited clinical use due to their potential adverse effects. In addition, due to the complex pathophysiological mechanisms of HF, there is a lack of therapeutic agents with specific efficacy in current clinical practice (Shan et al., 2019). Therefore, there is an increasing need to apply broader-ranging, safe, and effective drugs, especially those derived from natural products. In recent years, the low toxicity, multi-targeting, and multiple active ingredients of traditional Chinese medicine (TCM) have attracted increasing attention. TCM and its active ingredients (e.g., flavonoids, saponins, polysaccharides, alkaloids, etc.) can reduce liver damage by decreasing oxidative stress, inflammation, cell apoptosis, modulating immune function, and gut microbiota dysbiosis, thereby exerting an anti-HF effect (Shan et al., 2019; Ma et al., 2020; Hu et al., 2023; He et al., 2024; Yi-Wen et al., 2018).

Qinggan Yipi capsule (QgYp) is a hospital preparation from Suzhou Fifth People’s Hospital. It is composed of *Misgurnus anguillicaudatus* (Cantor) (abbreviated as *M. anguillicaudatus*, Niqiu in Chinese), *Indigo Naturalis* (Qingdai in Chinese), and *Bombyx Batryticatus* (Jiangcan in Chinese) in a ratio of 3:1:1. Among them, *M*. *anguillicaudatus* is the monarch medicine, and its natural component, *M*. *anguillicaudatus* polysaccharide, can ameliorate liver damage induced by CCl_4_, thioacetamide, and beta-naphthyl isothiocyanate in mice (Zhou et al., 2015). Additionally, freeze-dried *M*. *anguillicaudatus* powder also has a protective effect on liver damage caused by CCl_4_ (Zhang et al., 2021). *Indigo naturalis* is the minister medicine, with its main components being indigo red and indigo. Clinically, it is mainly used to treat ulcerative colitis. Its mechanisms primarily involve anti-inflammatory effects, promoting intestinal mucosal healing, regulating gut microbiome, and maintaining intestinal immune homeostasis (Ting et al., 2022; Xi et al., 2022). As an adjuvant medicine, *Bombyx batryticatus* has been proven to have a beneficial effect on hepatocellular carcinoma (Yuan et al., 2020). Its active ingredient, beauvericin, exhibits significant anti-tumor, antibacterial, antioxidant, and anti-inflammatory activities (Yv, 2023). In the clinical application process, we found that QgYp has the potential to improve liver fibrosis. However, the role of QgYp in improving HF and its potential mechanisms of action still need to be systematically elucidated.

In recent years, network pharmacology has become an important tool for revealing the connections between drugs, components, targets, and diseases, as well as for elucidating the mechanisms of drug action. In this study, we evaluated the anti-hepatic fibrosis effects of QgYp by developing a rat model of HF induced by CCl_4_. Additionally, we employed network pharmacology to prediction the potential mechanisms underlying the effects of QgYp. The findings suggest that the anti-fibrotic action of QgYp may be associated with the TGF-β signaling pathway. Building on this, the study further validated the antifibrotic mechanism of QgYp in regulating TGF-β1/Smad2/3 and gut microbiome imbalance through *in vitro* and *in vivo* experiments. The results indicate that the effect of QgYp in improving HF in rats may be related to the downregulation of the TGF-β1/Smad2/3 pathway, inhibition of HSCs activation, and regulation of gut microbiota dysbiosis. This offers a novel theoretical and experimental foundation for comprehensively ex-amining the antifibrotic mechanism and clinical application of QgYp.

## 2 Materials and methods

### 2.1 Preparation and quality control of the QgYp

QgYp is made by encapsulating the powders of *M. anguillicaudatus*, *Indigo Naturali*s, and *Bombyx Batryticatus* into capsule shells. Identified by Professor Yunzhi Pan from Suzhou Fifth People’s Hospital. It is a hospital preparation from Suzhou Fifth People’s Hospital (lot: 20220531). The recommended daily intake for humans is 9 capsules, with each capsule containing 220 mg of raw medicinal ingredients. All of its components are listed in Table 1. The quality control of QgYp has been published in previous studies (Sq, 2022). The content of oxalic acid should not be less than 0.80%, and the minimum content of indigo, indirubin, and protein should be 0.21%, 0.03% and 6.32% respectively.

**TABLE 1:** Composition of QgYp.

Chinese name	Latin name	Family	Weight(g)	Part used	Producting region
Niqiu	*Misgurnus anguillicaudatus (Cantor)*	Cobitidae	30	Entire body	Jiangsu
Qingdai	*Indigo Naturalis*	Acanthaceae	10	Leaves or stems	Jiangsu
Jiangcan	*Bombyx Batryticatus*	Bombycidae	10	Insect Body	Jiangsu

### 2.2 Reagents and antibodies

Colchicine (COL, 21FD) was obtained from Kunming Pharmaceutical Group Co. Ltd. (Kunming, China). Carbon tetrachloride (56-23-5) was obtained from Suzhou Chenze Chemical Co. Injection-grade olive oil (20211101) was obtained from Zhejiang Yutianshan Medicinal Oil Co. Ltd. (Quzhou, China). Primary antibodies to Smad2 (19245S), Smad3 (3700S), p-Smad2 (5339S), p-Smad3 (9523S), α-SMA (18338S), β-Actin (9520S), and the secondary antibodies Anti-rabbit IgG (7074S) and Anti-mouse IgG (7076S) were bought from Cell Signaling Technology (Boston, United States). TGF-β1 (ab215715), TGF-βR1 (ab235578) primary antibodies were purchased from Abcam (Cambridge, United Kingdom). Primary antibody type I collagen (Col I, bs-10423R) was bought from Beijing Bioss Company (Beijing, China). DMEM high glucose medium (2395973) was bought from Gibco (California, United States). Dimethyl sulfoxide (DMSO, 20150906) was bought from Tianjin Damao Chemical Reagent Factory (Tianjin, China). Tetramethyl azole salt (MTT, 70080125) was bought from Biosharp (Beijing, China). Fetal Bovine Serum (FBS, 11011-8611) was bought from Hangzhou Tianhang Biotechnology Co (Hangzhou, China). Trypsin (2594141) was bought from Shanghai Shining Crystal Biological Company (Shanghai, China). RIPA lysate (081022220930) was bought from Biyuntian Company (Shanghai, China). The ELISA kits for Hyaluronic Acid (HA, CSB-E08120r), Laminin (LN, CSB-E04646r), Procollagen Type III (PCIII, CSB-E15810r), Collagen Type IV (IV-C, CSB-E08883r) were bought from Wuhan Huamei Bioengineering Co Ltd. (Wuhan, China).

### 2.3 Animals and treatments

36 SPF-grade SD male rats (6–8 weeks, 200 ± 20 g) were obtained from Zhaoyan (Suzhou) New Drug Research Centre Co Ltd. (License No. SCXK (Su) 2018-0006). Our laboratory was kept in a properly ventilated situation with 12:12 h (L:D), relative humidity (45 ± 10)%, temperature (23 ± 2)°C, as well as unlimited access to sustenance and water. This study was conducted in accordance with the Chinese Guidelines for the Ethical Review of Laboratory Animal Welfare (GB/T 35892-2018) and Guidelines for the Care and Use of Laboratory Animals. All experiments were approved by the Ethics Committee of Suzhou Fifth People’s Hospital, with the Ethics Approval Number: (2021) Hospital Ethics Review Document (45).

After 1 week of acclimatization feeding, the rats were randomized into the control group (Con), the model group (Mod), the QgYp low-dose group (QgYpL), the QgYp medium-dose group (QgYpM), the QgYp high-dose group (QgYpH), and the colchicine group (COL), with six rats in each group. The Con group received intraperitoneal injections of an olive oil solution, while the other groups received intraperitoneal injections of a 40% CCl_4_ olive oil solution (CCl_4_ mixed evenly with olive oil in a volume ratio of 2:5), with an injection dose of 2 mL/kg, every 3 days for 8 weeks (Ziheng et al., 2023). Based on clinical dosages, the oral administration doses of QgYp and COL were determined using the equivalent dose coefficient conversion method from “Pharmacological Experimental Methodology,” which are 0.18 g/kg and 0.1 mg/kg, respectively (Luo et al., 2024). According to the “Handbook of Laboratory Animal Management and Practical Techniques,” the concentration gradient of QgYp was set at 0.18 g/kg, 0.9 g/kg, and 1.8 g/kg. Starting from the fifth week, different doses of QgYp and COL (prepared as a suspension using 0.5% CMC-Na) were administered via gavage at a dosage of 10 mL/kg, while the other groups received an equivalent dose of 0.5% CMC-Na once daily until the end of the eighth week. After fasting for 12 hours in the ninth week, an intraperitoneal injection of 2% pentobarbital sodium (3 mL/kg) is used to anesthetize rats. After blood is collected from the abdominal aorta, the rat is euthanized using the cervical dislocation method. Serum and liver tissues were collected for further observation.

**FIGURE 1:**
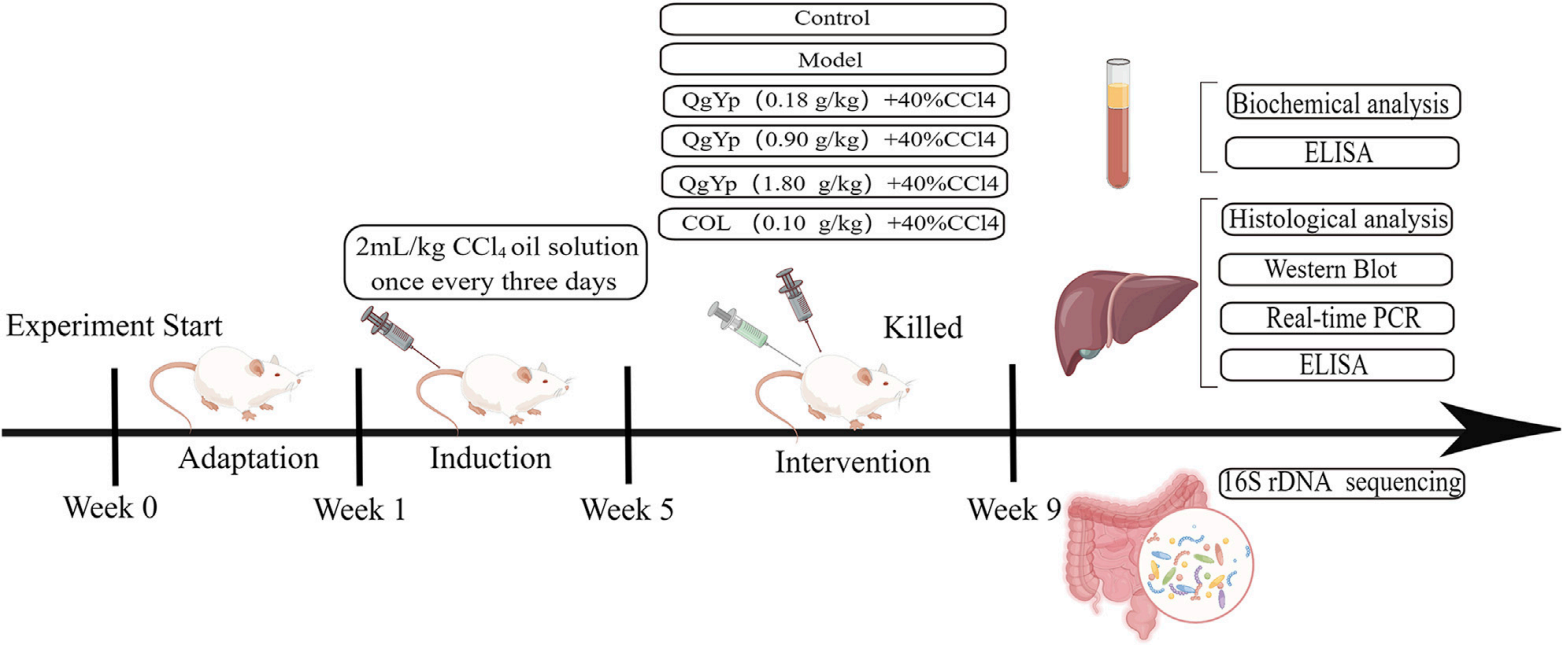
Diagram of the experimental protocol for the *in vivo* experiment.

24 SPF-grade SD male rats (6–8 weeks, 200 ± 20 g) were obtained from Zhaoyan (Suzhou) New Drug Research Centre Co Ltd. (License No. SCXK (Su) 2018-0006). The experimental environment and ethical principles are the same as those in the aforementioned experiments. After 1 week of acclimatization feeding, the rats were randomly divided into Con, Mod, and QgYp (0.9 g/kg) groups, with eight rats in each group. The modeling and administration methods are the same as those used in the above experiment. The modeling and administration methods are the same as those used in the above experiment. All rats were euthanized at the ninth week, and the cecal contents were collected, aliquoted into 1.5 mL Eppendorf tubes, and quickly frozen in liquid nitrogen. Figure 1 depicts the flowchart of the *in vivo* investigations.

### 2.4 Histopathological analysis

The right lobe of an intact rat liver was taken and fixed in 4% paraformaldehyde. After trimming, dehydrating, embedding in paraffin, sectioning, and deparaffinizing, hematoxylin-eosin (H&E) staining and Masson’s trichrome staining were performed, respectively. Calculate the ratio of collagen deposition area to the area of the field of view.

### 2.5 Serum biochemical analysis

Blood was drawn from the abdominal aorta of rats and allowed to stand for 2 hours. The supernatant was taken at 4°C, 3,000 r·min-1 for 10 min. An automatic biochemical analyzer measured blood levels of aspartate aminotransferase (AST) and alanine transaminase (ALT) to assess liver function.

### 2.6 Enzyme-linked immunosorbent test (ELISA)

According to the instructions of the ELISA assay kit, detect the content of HA, LN, PCIII, IV-C in the serum of the abdominal aorta.

### 2.7 Drug and disease-relevant target acquisition

According to the criteria of oral bioavailability (OB) ≥ 30% and drug-likeness (DL) ≥ 0.18, active compounds from Indigo naturalis were collected using the Traditional Chinese Medicine Systems Pharmacology Database (TCMSP, https://old.tcmsp-e.com/index.php). Use the “Traditional Chinese Medicine and Chemical Components Database” from the Chinese Academy of Sciences Chemical Database (www.organchem.csdb.cn) and the HERB database (http://herb.ac.cn/) to search for the active components of *M*. *anguillicaudatus* and *B. batryticatus*, and then use the SwissADME tool (http://www.swissadme.ch/) to further screen for active components that meet Lipinski’s rule. The potential targets of these active compounds were obtained through TCMSP and the Swiss Target Prediction analysis platform (http://www.swisstargetprediction.ch/). Subsequently, these targets were converted to standard gene names using the UniProt database (https://www.uniprot.org/).

Using “liver fibrosis” as the keyword, screen for human genes related to HF from GeneCards (https://www.genecards.org/) and the Online Mendelian Inheritance in Man (OMIM, https://omim.org/) databases. Then, use the VENNY 2.1.0 online platform (https://bioinfogp.cnb.csic.es/tools/venny) to visualize the intersection between the target compounds of QgYp and the HF-related genes.

### 2.8 Protein-protein interaction (PPI) network construction

Input the shared targets into the STRING database (https://cn.string-db.org/) to obtain the PPI file, with the protein type set to “*Homo sapiens*” and the minimum interaction threshold set to 0.4. Subsequently, utilize Cytoscape 3.10.2 software to construct the PPI network and sort the targets by degree.

### 2.9 GO functional enrichment and kyoto encyclopedia of genes and genomes pathway enrichment analysis

Import the common targets into the Metascape database (http://www.Metascape.org) for GO and KEGG analysis. Select the top 10 items with the smallest p-values for Biological Process (BP), Cellular Component (CC), and Molecular Function (MF) to create a bar chart. Additionally, select a portion of the KEGG signaling pathways to create a bubble chart. *P* < 0.05 is considered significant.

### 2.10 Preparation of drug-containing serum

30 healthy SD male rats were reared under normal conditions and arbitrarily divided into three groups: 12 rats were assigned to the control group, 12 to the QgYp group, and 6 to the COL group. The rats in the QgYp and COL groups received 1.8 g/kg of QgYp and 0.1 mg/kg of COL, respectively. The control group was given 0.5% CMC-Na once daily for 7 days. The procedure for giving gavage was repeated twice with a 2-h interval on the seventh day. Following the final dosage, blood was obtained, and the supernatant was centrifuged. These sera that we obtained were used in three parallel experiments.

### 2.11 Cell culture and cell viability assays

HSC-T6 cell lines (rat HSCs) were purchased from Wuhan Pricella Life Science and Technology Co. (Wuhan, China) and were continually grown in DMEM containing 10% FBS at 37°C in a humid environment with 5% CO_2_. Cells were separated, gathered, and injected into 96-well plates at 2 × 10^4^ cells/mL density when the cell concentration reached 80%–90%. Four groups of cells were created, containing a blank control group and three TGF-β1 concentration groups (5, 10, and 20 μg/L). Following incubation for 24 h, the original medium for culture was aspirated, and 200 μL of the corresponding medium was transferred to every well in each group, with a 24-h incubation period afterward. At the end of the intervention, the cell culture fluid was aspirated, and each well obtained 180 μL of medium containing DMEM and 20 μL of MTT solution (5 mg/mL), which were then incubated for 4 h. After shaking the wells for 10 min with 150 μL of DMSO solution added, a zymography (Molecular Devices in California, United States) was used to measure the absorbance at 450 nm.

### 2.12 Cell therapy

HSC-T6 cells were divided into the following groups after being implanted onto 96-well plates at a density of 2 × 10^4^ cells/mL, 200 μL wells: blank serum group, TGF-β1 group, TGF-β1+different concentrations of QgYp-containing serum group (5%, 10%, 15%, 20%), and TGF-β1+different concentrations of COL-containing serum group (5%, 10%, 15%, 20%), and the subsequent operation method is the same as that under “*Cell culture and cell viability assays*.”

### 2.13 Quantitative real-time polymerase chain reaction (qPCR)

Real-time quantitative polymerase chain reaction was used to analyze total RNA isolated from liver tissues and cells. Glyceraldehyde-3-phosphate dehydrogenase (GAPDH) and β-actin messenger RNA (mRNA) levels were utilized as internal controls. Primers for Smad2, Smad3, TGF-β1, GAPDH, Col I, α-SMA, TGF-βR1, and β-actin were designed and used. Quantitatively amplified primers are shown in the table below (Tables 2, 3).

**TABLE 2:** Primers for quantitative polymerase chain reaction of liver tissue.

Gene	Forward (5′-3′)	Reverse (5′-3′)
Smad2	GTT​TGC​CGA​GTG​CCT​AAG​TGA​TA	ACT​GTC​TGC​CTC​CGG​TAT​TCT​G
Smad3	CGA​GAA​CAC​TAA​CTT​CCC​CGC​T	GTG​GTT​CAT​CTG​GTG​GTC​GCT​A
TGF-β1	GCT​GAA​CCA​AGG​AGA​CGG​AAT​A	GCA​GGT​GTT​GAG​CCC​TTT​CC
GAPDH	CTG​GAG​AAA​CCT​GCC​AAG​TAT​G	GGT​GGA​AGA​ATG​GGA​GTT​GCT

**TABLE 3:** Primers for quantitative polymerase chain reaction of HSCs.

Gene	Forward	Reverse
Col I	CAT​CAA​GGT​CTA​CTG​CAA​CAT​GG	CAA​ACC​AGA​CAT​GCT​TCT​TCT​CC
α-SMA	ACC​ATC​GGG​AAT​GAA​CGC​TT	CTG​TCA​GCA​ATG​CCT​GGG​TA
Smad2	ATG​AGC​TCA​AGG​CGA​TCG​AG	AGA​ATC​TCT​GTG​TGC​CGA​GG
Smad3	CTT​ACA​AGG​CGG​CAC​ATT​GG	TTG​CAG​TTG​GGA​GAC​TGG​AC
TGF-βR1	TCA​GTC​ACC​GAG​ACC​ACA​GA	AGC​AAT​ACG​TAA​CTG​CCC​CT
β-actin	TGC​TAT​GTT​GCC​CTA​GAC​TTC​G	GTT​GGC​ATA​GAG​GTC​TTT​ACG​G

### 2.14 Western blot analysis

RIPA buffer with phenylmethylsulfonyl fluoride was used to lyse liver tissues and cells. After centrifugation at 12,000 r/min for 10 min, the supernatant was recovered. Total proteins were detected by BCA, an assay kit (Beyotime Biotechnology, Shanghai, China). On equal amounts of proteins, a 10% sodium dodecyl sulfate-polyacrylamide gel electrophoresis (SDS-PAGE) was carried out and electrotransferred to polyvinylidene fluoride (MERCK, New Jersey, United States). The membranes were closed in 5% skimmed milk for 2 h at room temperature, then incubated with primary antibody overnight at 4°C and secondary antibody for another 2 h at room temperature. Finally, immunoreactive bands were displayed using enhanced chemiluminescence. The data were normalized, and the relative expression of the target proteins was determined.

### 2.15 High-throughput 16S rRNA sequencing

The cecal contents of the rats were placed on dry ice and sent to Shanghai Majorbio Bio-pharm Technology Co., Ltd. for 16S rRNA sequencing. Following the directions on the FastDNA^®^ Spin Kit for Soil, DNA was extracted. Using primers for the variable region V3-V4 (338F-806R) of the 16S rRNA gene (Forward:5′-ACTCCTACGGGAGGCAGCAG-3′, Reverse: 5′-GGACTACHVGGGTWTCTAAT-3′) for PCR amplification. The PCR products were extracted and purified using a 2% agarose gel and quantified using a Synergy HTX (Biotek, United States). Library construction was performed using the NEXTFLEX Rapid DNA-Seq Kit (Bioo Scientific, United States). Sequencing was then performed on the MiSeq PE300 platform. The sequences were clustered into operational taxonomic units (OTUs) at a 97% similarity level. OTUs taxonomic classification was performed by comparing with the Silva 16S rRNA gene database (v138). Data analysis was conducted on the Majorbio Cloud platform (https://cloud.majorbio.com).

### 2.16 Statistical analysis

Statistical analysis was conducted using Graph Pad Prism 8 statistical software. All data are presented as mean ± standard deviation. Comparisons of intergroup data that meet the assumptions of normality and homogeneity of variance were performed using one-way analysis of variance (ANOVA), followed by Tukey’s post hoc analysis. For data that do not meet the normal distribution, the Kruskal-Wallis test was first used, followed by Dunn’s multiple comparison test. ANOSIM non-parametric tests were used to analyze whether the differences in microbial community structure between groups were significant. LEfSe analysis (Linear discriminant analysis Effect Size) was used to explore biomarkers of the gut microbiota. Spearman correlation analysis was used to assess the association between serum biochemical indicators and microbial species. A difference of *P* < 0.05 was considered statistically significant.

## 3 Results

### 3.1 QgYp ameliorated the CCl_4_-induced hepatic fibrosis in rats

**FIGURE 2:**
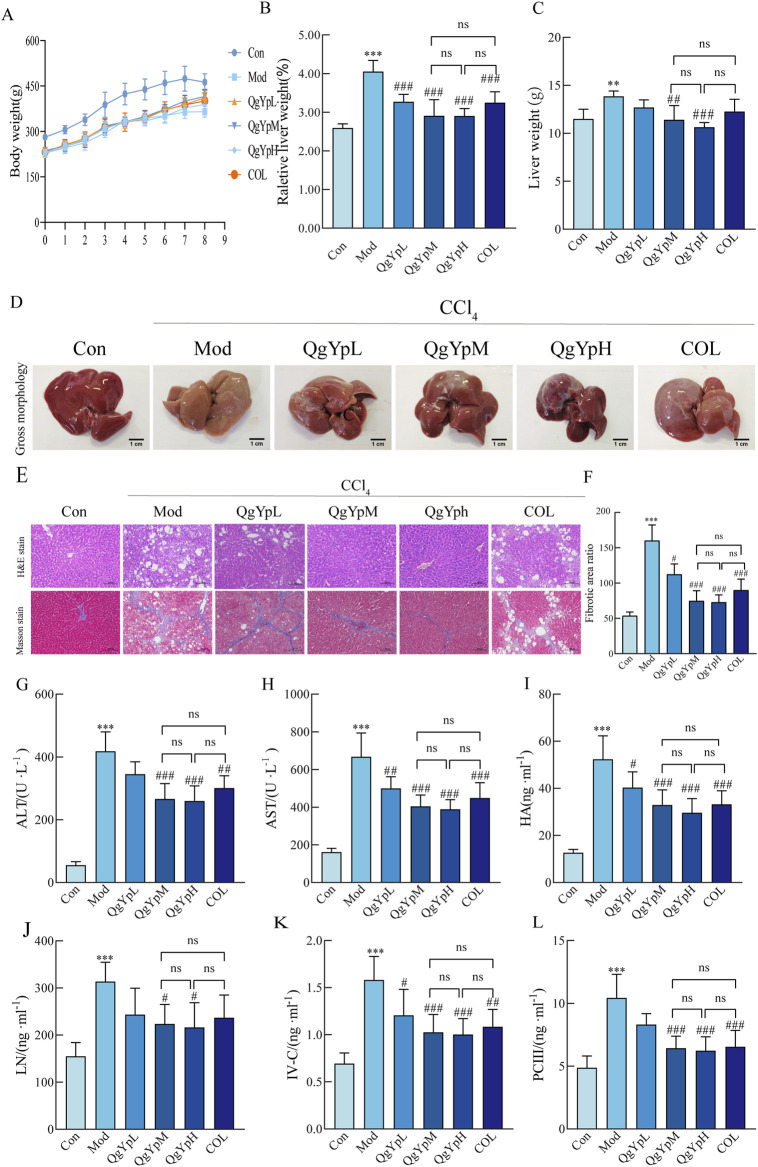
QgYp ameliorates CCl_4_-induced liver fibrosis in rats. **(A)** The growth trend of body weight in each group of rats (n = 6). **(B)** The liver coefficients of the various rat groups (n = 6). **(C)** The respective liver weights of the rat groups (n = 6). **(D)** Macroscopic features of liver morphology in each rat group. **(E)** Representative images of H&E staining and Masson staining in pathological liver sections of rats from each group. The magnification was ×200. **(F)** Comparison of the collagen deposition area deposition in liver tissue (n = 3). Effect of QgYp on CCl_4_-induced serum biochemical parameters in rats (n = 6), including **(G)** Alanine transaminase (ALT), **(H)** Aspartate aminotransferase (AST), **(I)** Hyaluronic acid (HA), **(J)** Laminin (LN), **(K)** Pre-collagen type III (PCIII), and **(L)** Collagen type IV (IV-C). Data are presented as mean ± standard deviation. **P < 0.01, ***P < 0.001 vs. control group; ^#^ *P* < 0.05, ^##^ *P* < 0.01, ^###^ *P* < 0.001 vs. model group.

In order to see how QgYp improves the liver of HF rats, we created HF models. Experimental and clinical studies have shown that COL has a therapeutic effect on liver fibrosis and is widely used as a positive control in anti-fibrosis research (Das et al., 2000; Kershenobich et al., 1988). Therefore, this study used COL to compare the effect of QgYp on improving liver fibrosis. Rats in all groups steadily gained weight during the rearing period. The hepatic index was somewhat decreased after treatment with different concentrations of QgYp and COL compared to the Mod group. Among them, the liver weight of QgYpM and QgYpH was significantly decreased, and there was no significant difference in the two groups’ hepatic index or liver weight. There was also no significant difference between these two groups compared to the COL group (Figures 2A–C). Next, we observed that the liver surface of the Con group was smooth, uniform in color, soft, and elastic in texture, with minimal adhesion to the surrounding tissues. In contrast, the liver surface of the Mod group was highly granular, dull in color, rigid, less elastic in texture, and heavily adherent to the surrounding tissues. Compared with the Mod group, the liver changes in CCl_4_-induced HF rats could be improved after the administration of QgYp and COL interventions, especially in the QgYpM, QgYpH, and COL groups. These groups exhibited reduced liver granularity, a more uniform color and a softer texture (Figure 2D). This indicates that QgYp could effectively improve liver status and liver coefficient in rats with liver fibrosis.

In addition, we observed QgYp’s impact on CCl_4_-induced pathomorphologic alterations associated with HF using H&E and Masson staining. Experiments using H&E staining revealed that rats in the Mod group had severe liver damage, characterized by disorganized hepatocyte arrangement, connective tissue hyperplasia, and lymphocyte infiltration. However, the intervention of QgYp and COL significantly ameliorated CCl_4_-induced liver injury. Masson staining revealed a significant increase in the collagen fiber area ratio in the rats of the Mod group, with a substantial amount of collagen fiber proliferation observed around the fusion area. However, the QgYp and COL interventions significantly reduced this pathological alteration, which showed no significant difference between the QgYpM and QgYpH groups and was not significantly different from that of the COL group (Figures 2E, F). This suggests that QgYp can effectively restore the histopathological changes in the liver of rats with liver fibrosis and inhibit the proliferation of collagen fibers in the liver tissue of rats with liver fibrosis.

Then, we observed QgYp’s impact on HF using biochemical assays and other techniques. As depicted in Figures 2G, H, the Mod group of rats had significantly higher ALT and AST serum levels. However, the intervention of QgYp and COL could significantly alter these changes compared to the Mod group. Furthermore, there was no distinction between the QgYpM and QgYpH groups, and there was no appreciable difference with the COL group. Meanwhile, the intervention of QgYp significantly reduced the levels of HA, LN, PC III, and IV-C in the serum of HF rats. These reductions were not different between the QgYpM and QgYpH groups and were not significantly different from the COL group (Figures 2I–L). This provides further evidence that QgYp can ameliorate liver injury and reduce the degree of fibrosis in rats with CCl_4_-induced liver fibrosis. In summary, QgYp significantly ameliorated CCl_4_-induced HF in rats.

### 3.2 Network pharmacology studies

#### 3.2.1 Collection and prediction of active ingredients of QgYp and HF-related targets

**FIGURE 3:**
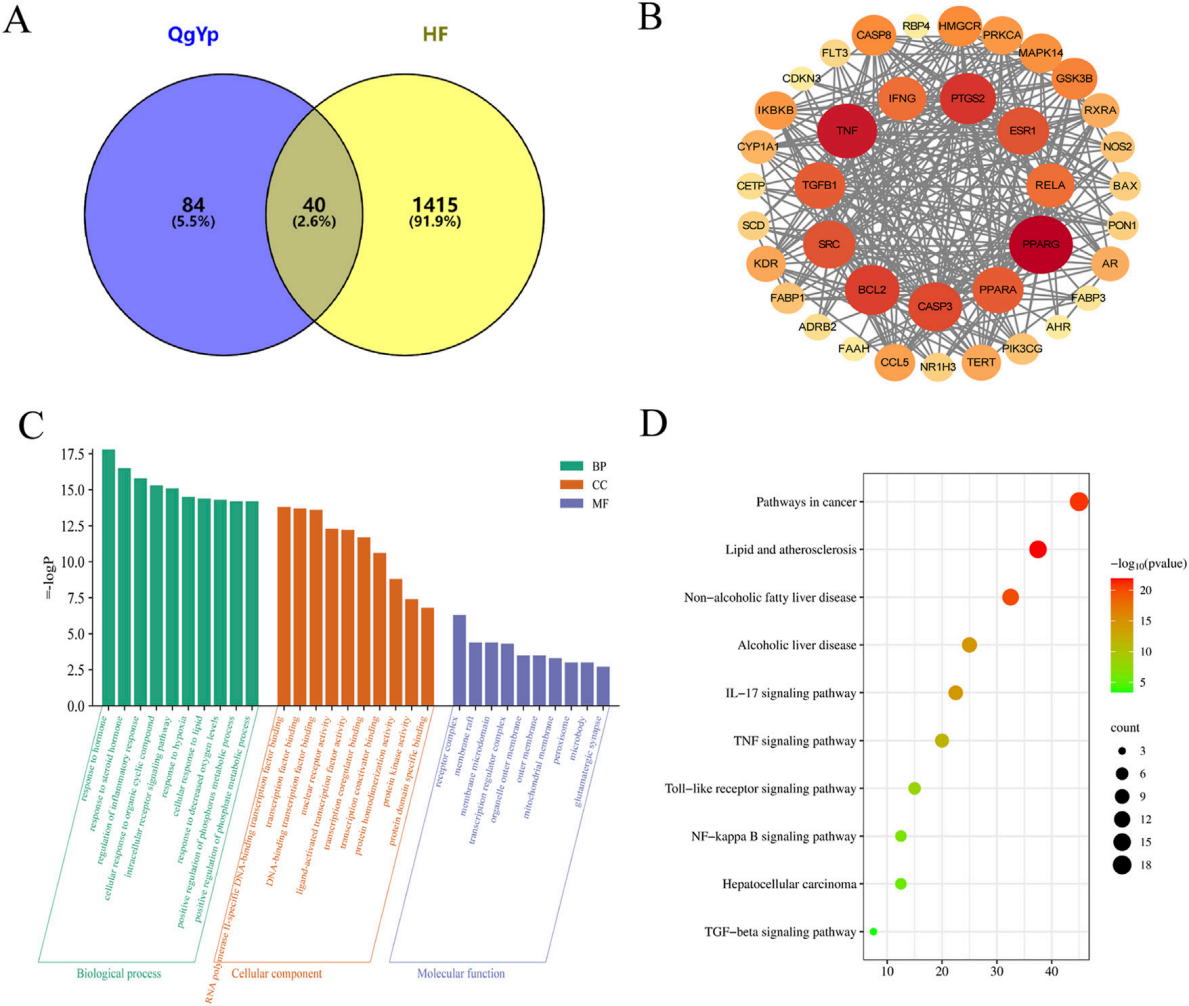
The drug-target-disease network, PPI network,GO and KEGG functional analysis. **(A)** The intersection targets of QgYp-related targets and HF-related targets. **(B)** PPI network diagram of targets related to HF treated with QgYp. **(C)** The top 10 of GO enrichment analysis in Biological processes (BP), cellular components (CC) and molecular function (MF). **(D)** The related signaling pathways were analyzed by KEGG.

We used TCMSP, the Chinese Academy of Sciences Chemistry Database, and the HERB database to obtain 59 potential active components of QgYp. After removing duplicate targets, we obtained 124 active component-related targets for QgYp from the online databases, along with 21 corresponding active components (Supplementary Table S1). In the Gene Cards and OMIM databases, 1455 HF-related targets were obtained. The correlation analysis of the relevant targets of HF and QgYp identified 40 potential targets of QgYp for the treatment of HF (Figure 3A). The intersecting targets were imported into the STRING database to obtain the PPI relationships. The selected 38 targets were analyzed in Cytoscape (Figure 3B). Using “degree,” “betweenness,” and “closeness” as limiting conditions, 11 core targets were filtered out, mainly including peroxisome proliferator-activated receptor gamma (PPARG), Tumor necrosis factor (TNF), Recombinant Prostaglandin Endoperoxide Synthase 2 (PTGS2), transforming growth factor-β1 (TGFβ1), and Recombinant V-Rel Reticuloendotheliosis Viral Oncogene Homolog A (RELA).

#### 3.2.2 GO functional enrichment and KEGG pathway analysis

In order to further understand the potential mechanism of QgYp in improving HF, we conducted GO annotation and KEGG pathway analysis on the intersecting targets (Figures 3C, D). GO analysis ranked the top ten BPs, CCs, and MFs based on the size of the P-values. The BPs mainly include regulation of inflammatory response, response to hormone, and response to molecules of bacterial origin. The CCs mainly include receptor complex, membrane raft, organelle outer membrane, and peroxisome. The MFs mainly include RNA polymerase II-specific DNA-binding transcription factor binding, nuclear receptor activity, protein kinase activity, and cytokine receptor binding. In addition, KEGG pathway enrichment analysis found that the therapeutic mechanism of QgYp in treating HF mainly involves the IL-7 signaling pathway, TNF signaling pathway, NF-κB signaling pathway, TGF-β signaling pathway (Figures 3C, D).

### 3.3 QgYp can downregulate the expression of the TGF-β1/Smad2/3 signaling pathway and inhibit HSCs activation

#### 3.3.1 QgYp downregulates the TGF-β1/Smad2/3 signaling pathway and inhibits the activation of HSCs in rat liver

Based on the results of network pharmacology, we found that QgYp can significantly affect the TGF-β signaling pathway, and the TGF-β1/Smad2/3 signaling pathway is one of the important signaling pathways that lead to HF. Therefore, we further observed the effect of QgYp on TGF-β1/Smad2/3 signaling pathway in HF rats.

**FIGURE 4:**
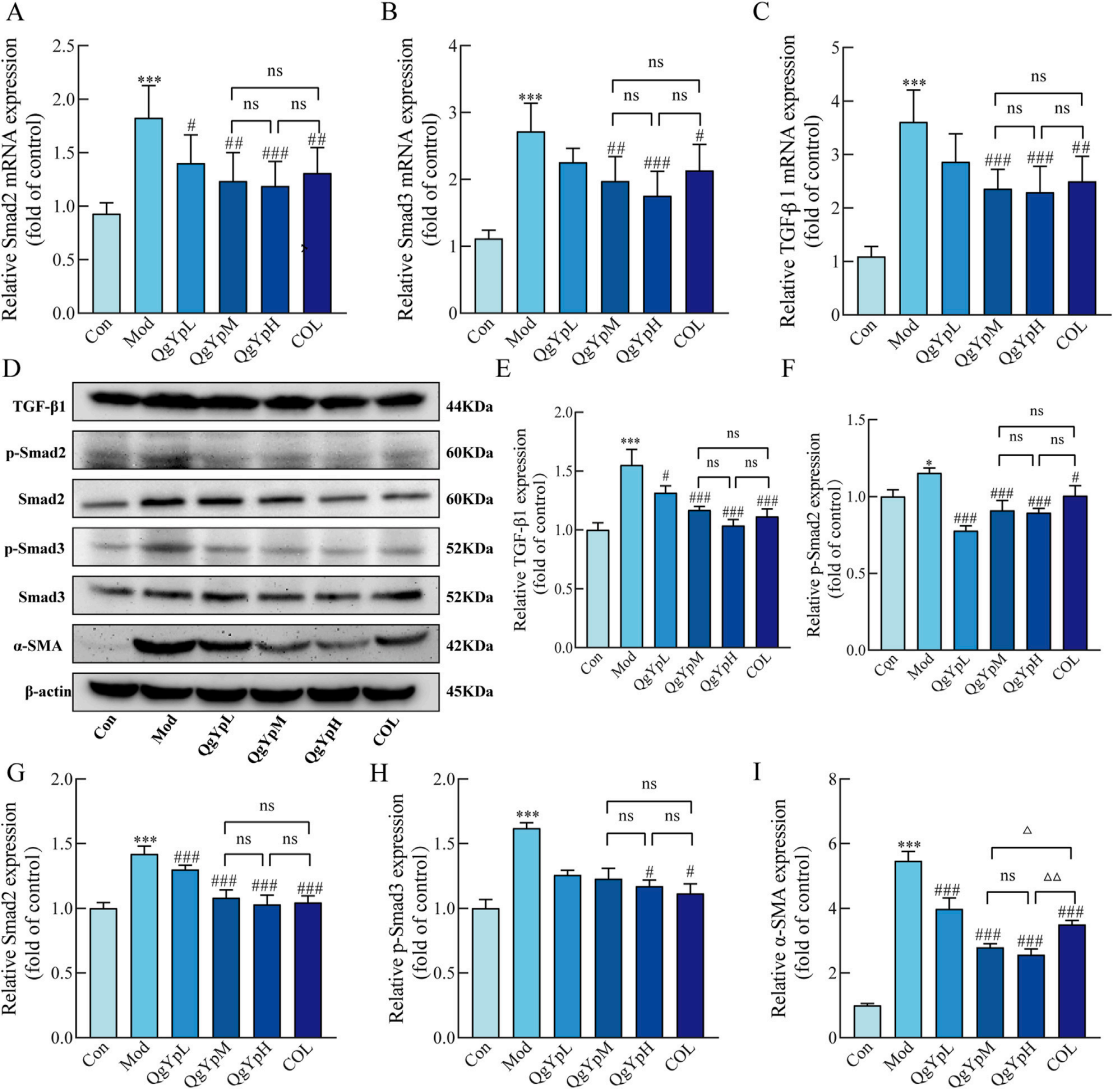
QgYp downregulates the TGF-β1/Smad2/3 signaling pathway and inhibits the activation of HSCs in rat liver. **(A–C)** Effects of QgYp on the expression of Smad2, Smad3, and TGF-β1 mRNA in the liver tissues of each group of rats (n = 6). **(D–I)** Effects of QgYp on the expression of the proteins TGF-β1, Smad2, p-Smad2, p-Smad3, and α-SMA in the liver tissues of each group of rats (n = 3). The mean ± standard deviation is used to express data. *P < 0.05, ***P < 0.001 vs. control group; ^#^ *P* < 0.05, ^##^ *P* < 0.01, ^###^ *P* < 0.001 vs. model group; ^Δ^ *P* < 0.05, ^ΔΔ^ *P* < 0.01 vs. COL group.

We evaluated the mRNA levels associated with the TGF-β1/Smad2/3 pathway using qPCR. The results indicated that the Mod group of rats had considerably higher Smad2, Smad3, and TGF-β1 mRNA expression in hepatic tissue than the Con group. However, the expression of these markers significantly decreased after the administration of QgYp and COL (Figures 4A–C). In addition, we employed Western blotting to further confirm the effects of QgYp and COL treatment on the protein expression levels associated with the TGF-β1/Smad2/3 signaling pathway. As shown in Figures 4D–I, the protein expressions of TGF-β1, Smad2, p-Smad2, p-Smad3, and α-SMA were significantly increased in the liver tissues of rats in the Mod group compared to those in the Con group. However, this increase was significantly decreased by the administration of QgYp and COL. Notably, there was no significant difference between the indicators mentioned above in the QgYpM and the QgYpH groups, and there was also no significant difference in the COL group. These results indicate that QgYp may improve liver fibrosis by downregulating the TGF-β1/Smad2/3 signaling pathway and inhibiting the activation of HSCs.

#### 3.3.2 QgYp can downregulate the TGF-β1/Smad2/3 pathway induced by TGF-β1 in HSC-T6 cells

**FIGURE 5:**
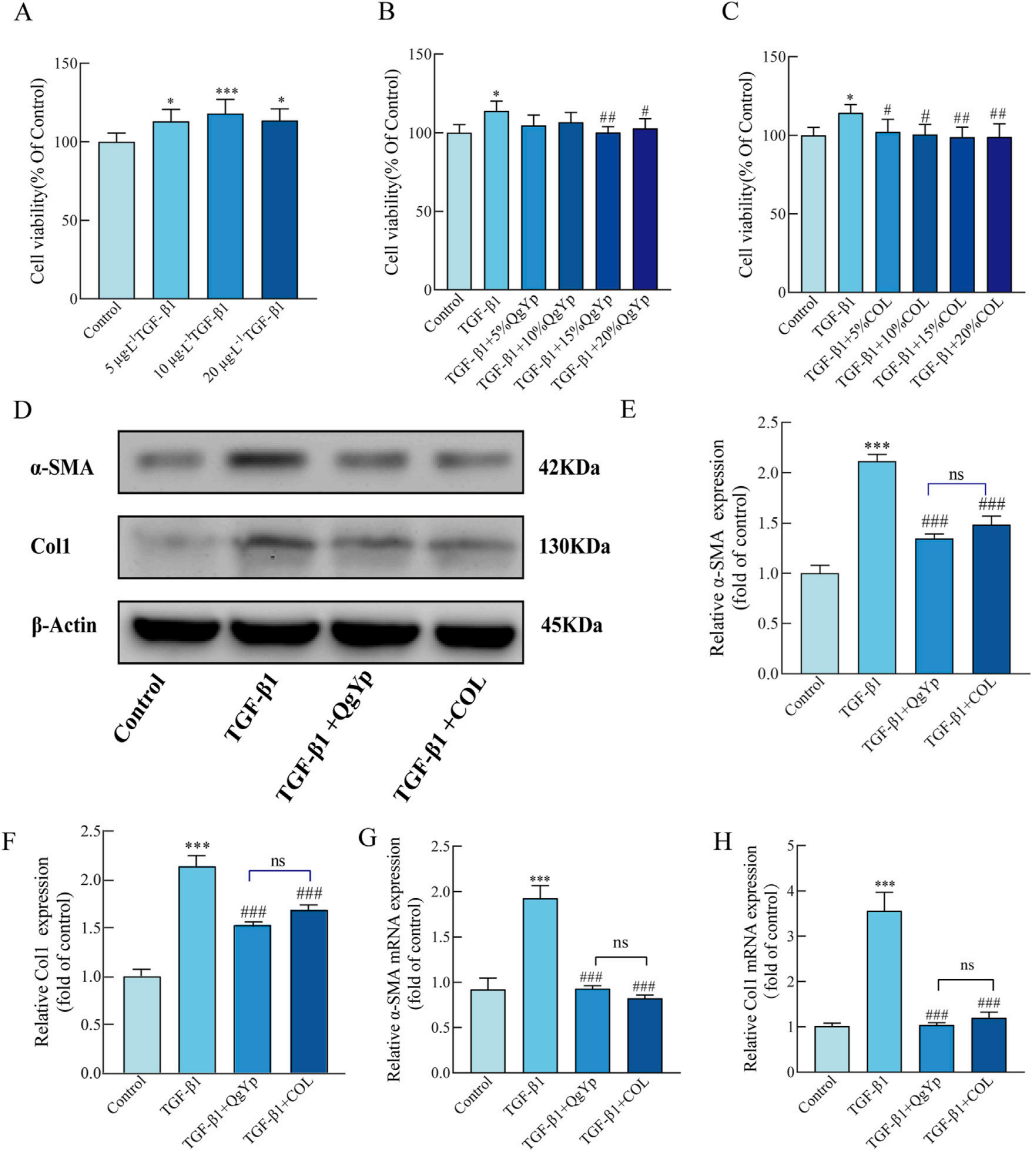
QgYp can inhibit TGF-β1-induced activation of HSC-T6 cells and downregulate the TGF-β1/Smad2/3 pathway. **(A)** Effects of various TGF-β1 concentrations on the growth of HSC-T6 cells. Effects of different concentrations of QgYp-containing serum **(B)** and COL-containing serum **(C)** on 10 μg L^-1^ TGF-β1-induced HSC-T6 cells. **(D–F)** Effects of QgYp on the levels of the proteins Col I and a-SMA in TGF-β1-induced HSC-T6 cells. **(G, H)** Effects of QgYp on TGF-β1-induced HSC-T6 cell a-SMA and Col I mRNA expression. The results are presented as the mean ± standard deviation of three parallel experiments. *P < 0.05, ***P < 0.001 vs. control group; ^#^ *P* < 0.05, ^##^ *P* < 0.01, ^###^ *P* < 0.001 vs. TGF-β1-induced group.

Next, we further study the mechanism by which QgYp inhibits TGF−β1-induced activation and proliferation of HSC-T6 cells by regulating the TGF−β1/Smad2/3 pathway. First, different concentrations of TGF-β1 (5 μg L^-1^, 10 μg L^-1^, and 20 μg L^-1^) were used to induce the proliferation of HSC-T6 cells. The results revealed that all three concentrations showed significant augmentative effects, with 10 μg L^-1^ yielding the most favorable outcome (Figure 5A). Subsequently, we exposed HSC-T6 cells induced by 10 μg L^-1^ TGF-β1 to different concentrations of drug-containing serum (QgYp and COL, 5%, 10%, 15%and 20%). As illustrated in Figures 5B, C, the proliferation of HSC-T6 cells was notably suppressed by the 15% and 20% QgYp-containing serum, as well as various concentrations of COL-containing serum, when compared to the TGF-β1-induced group. All of them inhibited proliferation best with 15% of drug-containing serum concentration. Therefore, we selected a 15% concentration of drug-containing serum as the intervention concentration for the subsequent study.

Subsequently, we evaluated the effect of QgYp on TGF-β1-induced activation of HSC-T6 cells. Increased expression of collagen type I (Col I) and ɑ-SMA are two characteristics of activated HSC-T6 cells, and TGF-β1 treatment increases their expression levels. According to qPCR and Western blotting results, 10 μg L^-1^ TGF-β1 significantly increased the Col I and ɑ-SMA mRNA and protein levels in HSC-T6 cells, and both QgYp and COL significantly reduced this effect. Moreover, the two groups had no discernible difference (Figures 5D–H). This indicates that QgYp can effectively inhibit the TGF-β1-induced HSC-T6 activation.

**FIGURE 6:**
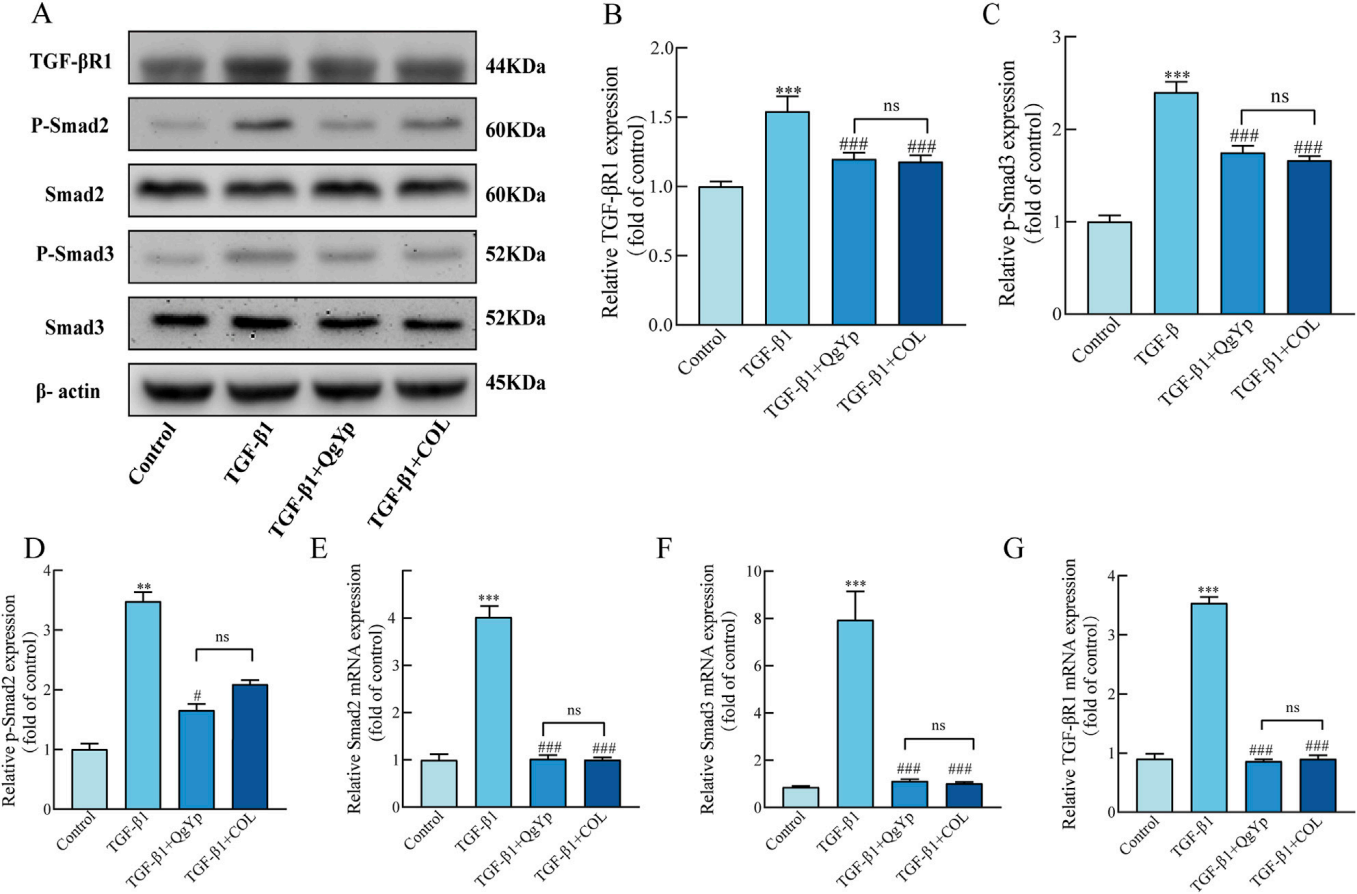
Effects of QgYp and COL on the TGF-β1/Smad2/3 pathway in activated HSC-T6 cells. **(A–D)** Effects of QgYp on the expression of the proteins TGF-βR1, p-Smad2, and p-Smad3 in TGF-β1-induced HSC-T6 cells. **(E–G)** Effects of QgYp on the mRNA expression of TGF-βR1, Smad2 and Smad3 in TGF-β1-induced HSC-T6 cells. The results are presented as the mean ± standard deviation of three parallel experiments. **P < 0.01, ***P < 0.001 vs. control group; ^#^ *P* < 0.05, ^###^ *P* < 0.001vs TGF-β1-induced group.

Finally, we determined the impact of QgYp and COL on the TGF-β1/Smad2/3 signaling pathway in activated HSC-T6 cells. Western blotting results revealed that TGF-β1-induced greatly enhanced the expression of the related proteins TGF-βR1, p-Smad2, and p-Smad3 in HSC-T6 cells compared to the control group. In contrast, both QgYp and COL were able to significantly decrease the expression of related proteins (Figures 6A–D). According to subsequent qPCR data, the TGF-βR1, Smad2, and Smad3 mRNA expression was considerably higher in the TGF-β1 group. However, both QgYp and COL were able to reduce the expression of the related mRNAs significantly. Additionally, there was no discernible difference between QgYp and COL in the values of the indicators above (Figures 6E–G). According to this, QgYp may prevent HSC-T6 cells from activating and proliferating by suppressing the expression of the TGF-β1/Smad2/3 pathway.

In the *in vivo* animal experiments, we observed a significant reduction in liver fibrosis in the QgYp treatment group of rats, accompanied by improvements in liver function indicators. Further research revealed that the QgYp intervention can significantly decrease the expression of the TGF-β1/Smad2/3 signaling pathway and inhibit the activation of HSCs in the rat liver. This finding is consistent with the results of *in vitro* cell experiments. We observed a significant decrease in the proliferation capacity of HSC-T6 cells after QgYp treatment, further confirming the potential of QgYp to inhibit HSCs activation. Molecular mechanism studies further demonstrated that QgYp treatment can significantly reduce the expression of the TGF-β1/Smad2/3 signaling pathway, leading to the inhibition of HSC-T6 activation, consistent with the results of *in vivo* experiments.

In summary, we find that QgYp can treat HF, and its action may be related to inhibiting the TGF-β1/Smad2/3 pathway and suppressing HSCs activation and proliferation.

### 3.4 QgYp regulates the overall structure of the gut microbiota in rats with liver fibrosis

We employed 16s rDNA sequencing to evaluate the impact of QgYp on alterations in the gut microbiota in rats suffering from CCl_4_-induced HF. Previously, we learned that COL has more significant gastrointestinal toxicity and can disrupt intestinal homeostasis to aggravate intestinal toxic load (Dong et al., 2022). Therefore, we did not use COL as a positive control in our experiment to observe the regulation of gut microbiome by QgYp. In addition, In previous studies, we found that there was no significant difference in the effects of medium and high doses of QgYp, indicating that the medium dose can serve as a basis for further research on the quantitative relationship of the drug in future studies. Therefore, in this part of the study, we chose the medium dose as the research dosage.

**FIGURE 7:**
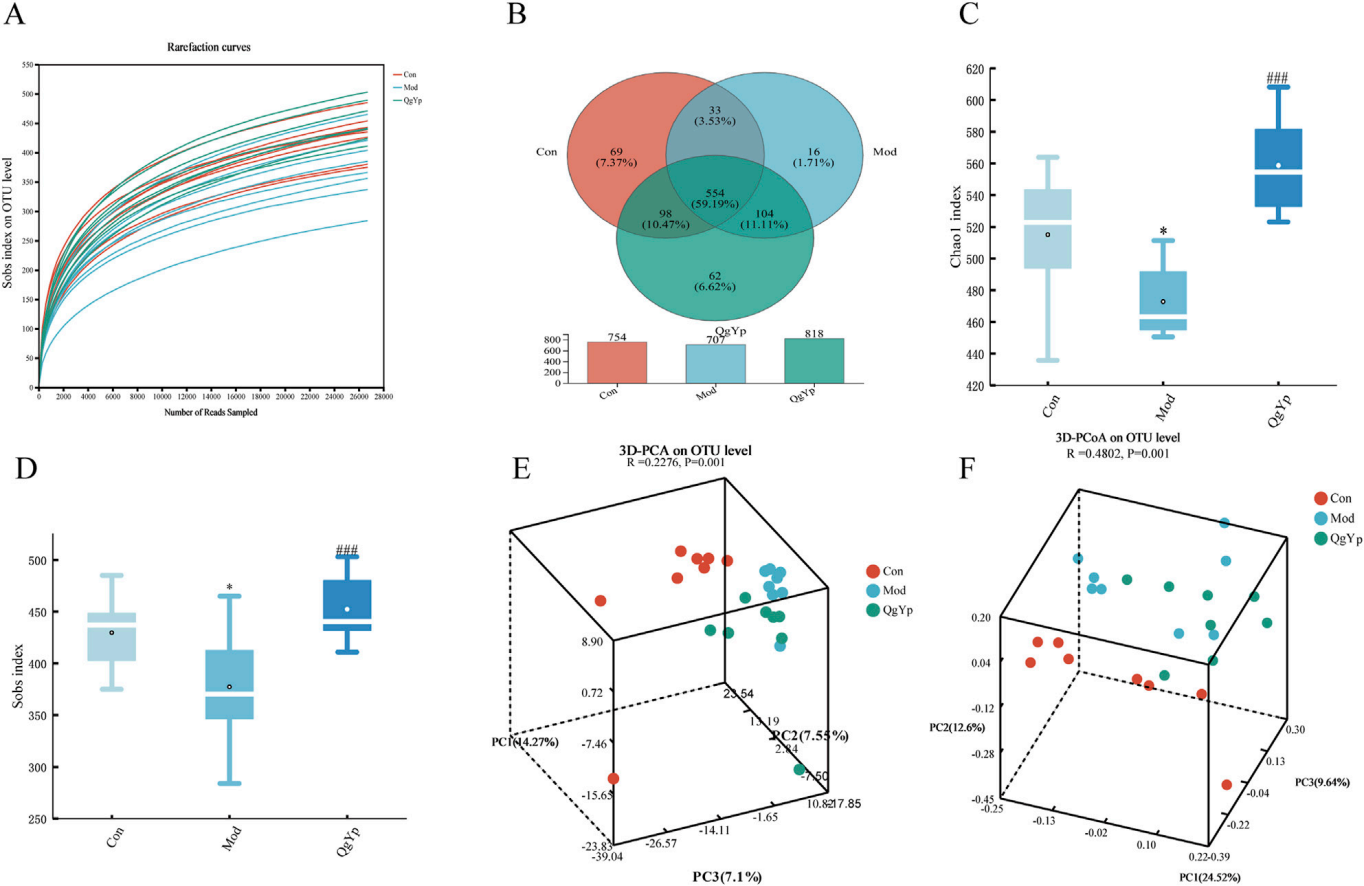
QgYp alters the overall structure of the gut microbiota in rats with liver fibrosis (n = 8). **(A)** Rarefaction curve. The vertical coordinate represents the number of species observed, while the horizontal coordinate represents the data extracted. **(B)** Wayne diagram of species. Numbers that overlap indicate species shared by several groups, while numbers that do not indicate species specific to the associated group. **(C, D)** Alpha diversity analysis. The horizontal coordinate represents the subgroup’s name, while the vertical coordinate represents the value of the alpha diversity index (Chao1 and Sobs). **(E, F)** Beta diversity analysis. Principal components analysis (PCA) and principal coordinates analysis (PCoA) were analyzed based on the OTU level. *P < 0.05 vs. control group; ^###^ *P* < 0.001 vs. model group.

As shown in Figure 7A, the rarefaction curve indicated that all samples had sufficient sequencing depth. Operational taxonomic units (OTUs) with a 97% similarity level were used for clustering. The Wayne diagram revealed that were 554 OTUs shared among the three groups. Furthermore, the number of OTUs in the Mod group decreased compared to the Con group. However, QgYp significantly increased the number of OTUs (Figure 7B). Subsequently, we utilized the Chao1 and Sobs indices to evaluate the abundance and variety of gut microbiome communities (Figures 7C, D). Compared to the Con group, the Chao1 and Sobs indexes’ values were significantly lower in the Mod group. However, after the QgYp intervention, both the Chao1 index and Shannon index values significantly increased compared to the Mod group. This finding was consistent with the Wayne plot results, suggesting that QgYp intervention improved the richness and diversity of gut microbiome in HF rats. Next, we utilized principal component analysis (PCA) and principal coordinate analysis (PCoA) to illustrate that the gut microbiome changed the three groups (Figures 7E, F).

**FIGURE 8:**
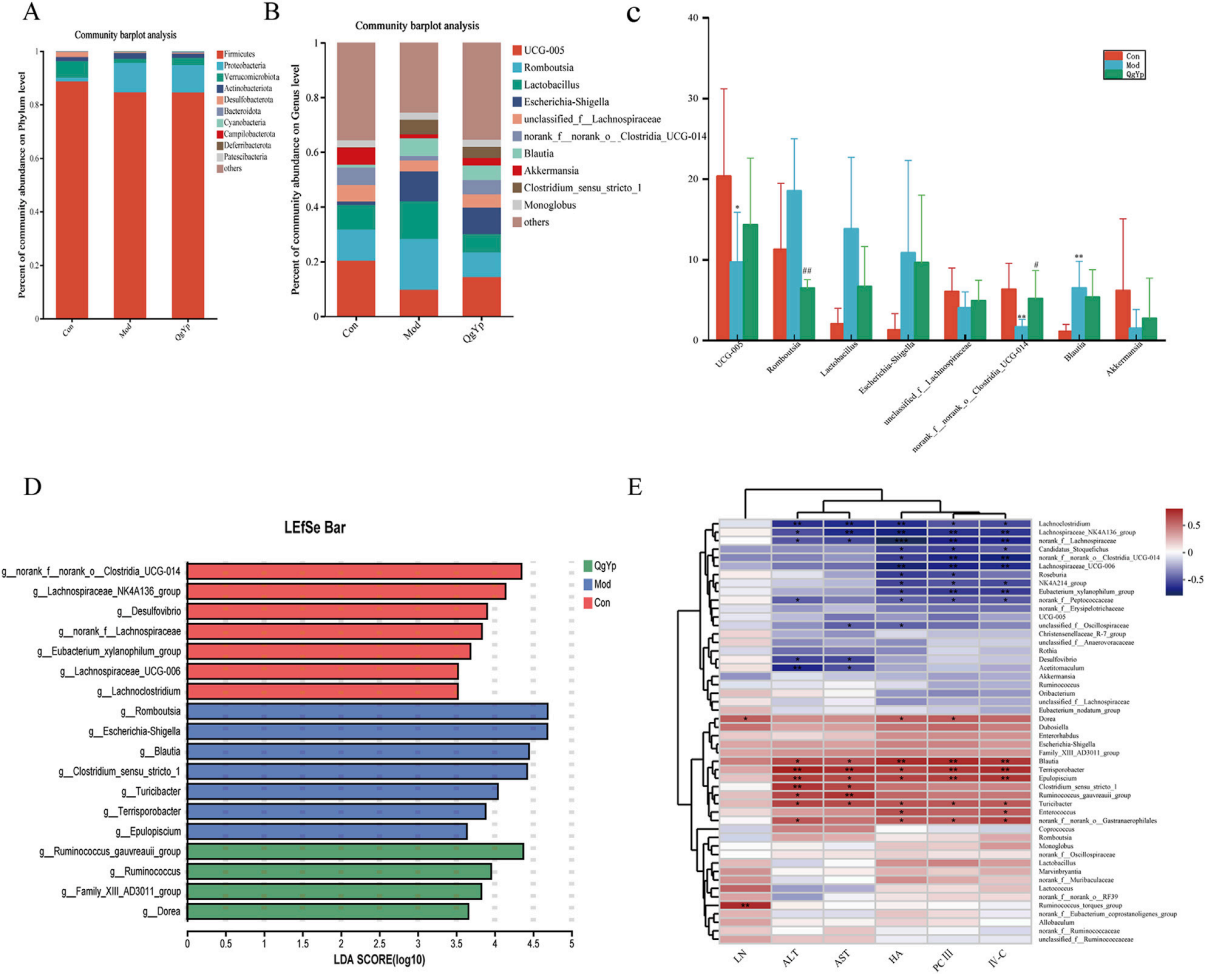
Effect of QgYp on changes in microbial community composition at the phylum and genus levels (n = 8). **(A)** Representative histogram of gut microbiota at the phylum level. **(B)** Representative histogram of the gut microbiota at the genus level. **(C)** Detailed analysis of the eight most abundant species at the genus level; and **(D)** LDA discrimination histogram. The LDA threshold was 3.5. **(E)** Correlation heatmap. Serum biochemical indicators and species served as the horizontal and vertical coordinates, and the correlation R and P values were calculated. *P < 0.05, **P <0 .0.01, ***P <0 .0.001.

We also observed the changes in the composition of the microbial community at the phylum and genus levels in each group of rats to better understand the impact of QgYp on the gut microbiome. First, detailed analysis at the phylum level showed that *Firmicutes*, *Proteobacteria*, and *Verrucomicrobiota* accounted for more than 95% of the total bacteria. The relative abundance of *Firmicutes* and *Verrucomicrobiota* reduced, while that of *Proteobacteria* increased in the Mod group compared to the Con group. However, QgYp intervention altered the relative abundance of *Verrucomicrobiota* and *Proteobacteria* (Figure 8A). Subsequent detailed analyses at the genus level showed that *UCG-005*, *Romboutsia*, *Lactobacillus*, *Escherichia-Shigella*, *unclassified_f__Lachnospiraceae, norank_f__norank_o___Clostridia_UCG-014*, *Blautia*, and *Akkermansia* as the top eight species in relative abundance. Overall, the treatment with CCl_4_ either increased or decreased the relative abundance of these eight bacteria. However, the intervention of QgYp could partially alter these changes. Among them, the Mod group exhibited a significantly higher relative abundance of *Blautia*, while the relative abundance of *UCG-005* and *norank__f__norank__o_Clostridia_UCG-014* was notably lower than that of the Con group. Notably, compared to the Mod group, QgYp significantly higher the abundance percentage of *norank_f__norank__o___Clostridia_UCG-014*. Furthermore, QgYp therapy significantly decreased *Romboutsia* abundance (Figures 8B, C).

In addition, Then, we performed LefSe analysis to pinpoint statistically significant biomarkers and the dominant microbiota in each group. Con group for *norank_f__norank__o_Clostridia_UCG-014*, *Lachnospiraceae_NK4A136_group*, *Desulfovibrio*, *the norank__f__Lachnospiraceae*, *Eubacterium_xylanophilum_group*, *Lachnospiraceae_UCG-006*, *Lachnoclostridium*; Mod group for *Romboutsia*, *Escherichia- Shigella*, *Blautia*, *Clostridium_sensu_stricto_1*, *Turicibacter*, *Terrisporobacter*, *Epulopiscium*; QgYp group for *Ruminococcus_gauvreauii_group, Ruminococcus*, *Family_XIII_AD3011_group*, *Dorea* (Figure 8D). These findings suggest that QgYp may have a role in regulating the gut microbiota.

Correlation heat map research results revealed that *Lachnoclostridium*, *Lachnospiraceae_NK4A136_group*, *norank__f__Lachnospiraceae* were significantly negatively correlated with ALT, AST, HA, PCIII, and IV-C; and *Candidatus_ Stoquefichus*, *norank _f_norank_o_Clostridia_UCG-014*, *Lachnospiraceae_UCG-006*, *NK4A214_groupEubacterium_ xylanophilum_group*, *norank__f__ Peptococcaceae* showed significant negative correlation with HA, PCIII, IV-C; *Desulfovibrio*, *Acetitomaculum* showed a significant negative correlation with ALT, AST. While *Blautia*, *Terrisporobacter*, and *Epulopiscium* showed a significant positive correlation with ALT, AST, HA, PCIII, and IV-C, and *Dorea* and *Ruminococcus_torques_group* showed significant positive correlation with LN (Figure 8E). In conclusion, the mechanism by which QgYp improves liver fibrosis may be related to its regulation of the abundance, diversity, and structure of the gut microbiota.

## 4 Discussion

QgYp is clinically safe as a hospital preparation that has been used for a long time in the clinic, primarily for the treatment of chronic hepatitis B (Sl, 2002). In the present study, we demonstrated the ameliorative effect of QgYp in HF rats using a CCl_4_-induced rat model of HF, which was comparable to the efficacy of the widely used therapeutic drug COL. Subsequently, network pharmacology results showed that the potential mechanism of action of QgYp to improve HF is related to TGF-β signaling pathway. Further experimental findings showed that the anti-fibrotic mechanism may be related to the downregulation of TGF-β1/Smad2/3 signaling pathway expression, inhibition of HSCs activation, and regulation of gut microbiome imbalance.

In the present study, CCl_4_-induced HF model in rats was used. The model is suitable for the study of human liver fibrosis due to its good reproducibility and the fact that it induces hepatocellular damage similar to the cellular changes seen in human liver disease (Wang et al., 2021; Li et al., 2021). In preliminary studies, we found that CCl_4_-induced liver fibrosis in rats formed by the end of 4 weeks, and after stopping CCl_4_ injection for 4 weeks, the model showed a self-healing phenomenon. Therefore, the duration of treatment should not exceed 4 weeks (Ziheng et al., 2023). In this experiment, to avoid interference from self-healing, we administered the treatment simultaneously with CCl_4_ injection to enhance the credibility and scientific validity of the results.

HF can lead to swelling of the liver and cause pathologic changes in the liver as well as hepatocyte damage. When hepatocytes are damaged, ALT and AST are released from the cells into the bloodstream, so both are often used as clinical markers of hepatocyte injury. In addition, significantly increased serum concentrations of HA, LN, PCIII, and IV-C are often used as a primary indicator for the assessment of HF (Bailey et al., 2012; Ma et al., 2021). In this study, we found that CCl_4_ induction caused significant pathological injury to rat liver tissue and significantly increased the levels of ALT, AST, HA, LN, IV-C and PC III. In contrast, QgYp treatment effectively alleviated the pathological injury of liver tissue and decreased the levels of ALT, AST, HA, LN, IV-C and PC III. This suggests that QgYp can effectively improve CCl_4_-induced HF in rats.

Network Pharmacology is an important tool for revealing the complex mechanisms of traditional Chinese medicine formulas. In this study, we screened 40 intersecting targets between drugs and diseases. A PPI network was constructed for these intersecting targets, and the main gene functions were analyzed using GO and KEGG analyses. The results revealed that genes such as PPARG, TNF, PTGS2, TGF-β1, and RELA may be the core targets of QgYp for the treatment of HF, and found that the pathways through which QgYp acted on HF included IL-7 signaling pathway, TNF signaling pathway, NF-κB signaling pathway, and TGF-β signaling pathway. Activation of HSCs is a key factor leading to HF, and TGF-β1, as one of the most potent pro-fibrotic cytokines in the TGF-β family, can function together with Smad2 and Smad3 to cause fibrosis (Feng et al., 2024). Therefore inhibiting the activation of HSCs through the TGF-β1/Smad2/3 pathway may be a key mechanism for QgYp to ameliorate HF. In addition, the regulation of gut microbiome is closely related to the screened core drug-disease targets. For example, PPAR signaling pathway is mainly involved in lipid metabolism in the liver, Xiao Y et al. found that the interaction between drugs and gut microbiome can affect PPAR signaling pathway in the liver (Xiao et al., 2024). Zhang L et al. found that regulating the gut microbiome can increase the plasma secondary bile acids and thus inhibit the liver injury caused by the TNF pathway (Zhang et al., 2024). When the gut microbiome imbalance is resolved, the HSCs activation and proliferation as well as ECM overproduction can be inhibited by inhibiting the TGFβ/Smad signaling pathway (Zhang et al., 2017). Therefore, regulating gut microbiome imbalance may be an important way for QgYp to ameliorate liver fibrosis.

TGF-β1 is released after liver injury and binds to the TGF-β type II receptor on the hepatocyte membrane. This interaction leads to the recruitment and phosphorylation of the TGF-β receptor type I, forming a complex. This complex stimulates the phosphorylation of Smad2 and Smad3 (Qiu-Shi and Ping, 2017). Finally, a complex of p-Smad2, p-Smad3, and Smad4 enters the nucleus and controls gene transcription, increasing the creation of ECM and promoting the activation of HSCs that result in HF (Jingru, 2021). In addition, Col I and the α-SMA protein are significantly upregulated in activated HSCs, with the α-SMA protein as a critical indicator of HSCs activation (Baghaei et al., 2022; Kamm and Mccommis, 2022; Sufleţel et al., 2021; Yang et al., 2021; Liao et al., 2022; Luo et al., 2022). Our results showed that QgYp could downregulate TGF-β1/Smad2/3 pathway-related mRNA and protein expression and downregulate the production of the α-SMA protein in rat liver. This suggests that QgYp may play a role in ameliorating HF by down-regulating of the TGF-β1/Smad2/3 signaling pathway and inhibiting HSCs activation. Based on this, we prepared serum containing QgYp acting on TGF-β1-induced HSC-T6 cells. The results showed that the expression of mRNAs and proteins related to α-SMA, Col I, and the TGF-β1/Smad2/3 pathway was downregulated in HSC-T6 cells after QgYp treatment. These findings suggest that QgYp can improve liver fibrosis, and the mechanism may be related to the downregulation of the TGF-β1/Smad2/3 pathway and the inhibition of the activation and proliferation of HSCs. In follow-up studies, we can futher clarify the regulatory mechanism by applying inhibitors or neutralizing antibodies to the TGF-β1/Smad2/3 pathway in HSC-T6 cells.

The intestine and liver are anatomically inseparable and functionally interrelated, leading to a complex interaction between gut microbiome dysbiosis and the occurrence and progression of liver fibrosis (Liu Y. T. et al., 2021). It has been reported that TMC can effectively prevent the occurrence and progression of liver fibrosis by regulating the gut microbiota and improving intestinal barrier dysfunction (Xue et al., 2024). For example, phenylethanol glycosides from Cistanche tubulosa can prevent rat liver fibrosis induced by bovine serum albumin through the gut microbiota (Qi et al., 2025); Si-Ni-San can effectively alleviate liver injury by regulating gut microbiota dysbiosis and enhancing intestinal barrier function (Li et al., 2025). In this study, We investigated whether QgYp’s function in HF development partially relies on gut microbiome regulation using 16S rRNA gene sequencing. At the phylum level, the relative abundance of *Firmicutes* and *Verrucomicrobiota* decreased, and the abundance of *Proteobacteria* increased in the Mod group compared to the Con group. However, the intervention of QgYp changed the relative abundance of *Verrucomicrobiota* and *Proteobacteria*. Notably, at the genus level, the intervention of QgYp partially reversed the changes in the top eight genera in terms of abundance. Among these, QgYp significantly increased the abundance of *norank_f__norank__o___Clostridia_UCG-014* and significantly decreased the abundance of *Romboutsia*. One study discovered a negative correlation between liver injury and *g__norank_f__norank__o_Clostridia_UCG-014,* which is the same as our findings (Zhuge et al., 2022). In addition, correlation analysis between liver function indices, liver fibrosis indices and species of Top50 showed a highly negative correlation between *norank_f__norank__o___Clostridia_UCG-014* and HA, PCIII, and IV-C concentrations. In summary, QgYp regulated the gut microbiome dysbiosis in HF rats, and *g__norank_f__norank__o_Clostridia_UC*G-014 may be a kay factor in the amelioration of HF in rats by QgYp.

Although this study did not elucidate the interaction between the TGF-β1/Smad2/3 pathway and gut microbiota in liver fibrosis, we found that intervention with QgYp can reduce the levels of Lipopolysaccharide (LPS) in the liver, portal vein serum, and colon of rats (Supplementary Table S1). LPS is an important component of the cell wall of Gram-negative bacteria. When dysbiosis occurs, it can pass through the damaged intestinal barrier, enter the liver through the portal vein, and directly bind to the TLR4 receptor on HSCs, leading to the activation and differentiation of HSC (Liu et al., 2024). Activated HSCs release various matrix proteins and cytokines, such as TGF-β, collagen, etc. These molecules promote the generation and deposition of fibrous connective tissue in the liver, thereby advancing liver fibrosis (Carpino et al., 2020). We will also continue to explore the relationship between the two in liver fibrosis in future. In addition, due to the mechanism of QgYp metabolism in the gastrointestinal tract and its interaction with gut microbiome is highly complex, the causality of the gut microbiome for the inhibition of HF by QgYp via gut microbiome needs to be verified by experiments such as fecal transplantation. The effective components of TMC formulas are relatively complex, and these active ingredients often play a crucial role in the treatment of diseases. Therefore, studying the anti-liver fibrosis mechanisms of QgYp’s active components is an important way to reveal its potential therapeutic effects. Nevertheless, our findings provide important preliminary information on the role of QgYp in the treatment of liver fibrosis and suggest that QgYp may be a promising candidate for the treatment of liver fibrosis.

## 5 Conclusion

In conclusion, our research unequivocally showed that QgYp improves HF. Its effects might be intimately linked to the suppression of the TGF-β1/Smad2/3 signaling pathway and the regulation of the balance of the gut microbiome. This offers a novel theoretical and experimental foundation for comprehensively examining the antifibrotic mechanism and clinical application of QgYp.

## Data Availability

The data presented in this study have been deposited in the NCBI database. Accession numbers: PRJNA1183351; PRJNA1183342. Links: https://www.ncbi.nlm.nih.gov/bioproject/PRJNA1183351; https://www.ncbi.nlm.nih.gov/bioproject/PRJNA1183342.

## Abbreviations

QgYp, Qinggan Yipi Capsule; HF, Hepatic fibrosis; CCl_4_, Carbon tetrachloride; HSC-T6, Rat Hepatic Stellate Cell Line; ELISA, Enzyme-Linked Immunosorbent Assay; TGF-β1, Transforming growth factor β1; ALT, Alanine aminotransferase; AST, Aspartate aminotransferase; HA, Hyaluronic acid; LN, Laminin; PCIII, Type III procollagen; IV-C, Type IV collagen; qPCR, Quantitative Polymerase Chain Reaction; Smad2, Mothers Against Decapentaplegic Homolog 2; p-Smad2, Phosphorylated Mothers Against Decapentaplegic Homolog 2; p-Smad3, Phosphorylated Mothers Against Decapentaplegic Homolog 3; α-SMA, Alpha Smooth Muscle Actin; TCM, Traditional Chinese medicine; ECM, Extracellular Matrix; HSCs, Hepatic stellate cell; Mod, Model group; QgYpL, low-dose group, QgYpM, medium-dose group, the; QgYpH, high-dose group; H&E, hematoxylin-eosin.
