# Supplementary material for: Qinggan Yipi capsule ameliorates hepatic fibrosis in rats by down-regulating the TGF-β1/Smad2/3 signaling pathway and improving gut microbiota imbalance
Source: Front Pharmacol. 2025 Jan 24;16:1525914. doi: 10.3389/fphar.2025.1525914 (PMC11802500; doi:10.3389/fphar.2025.1525914)
Supplement: Supplementary file 1 [file DataSheet2.pdf]

TGF-β1

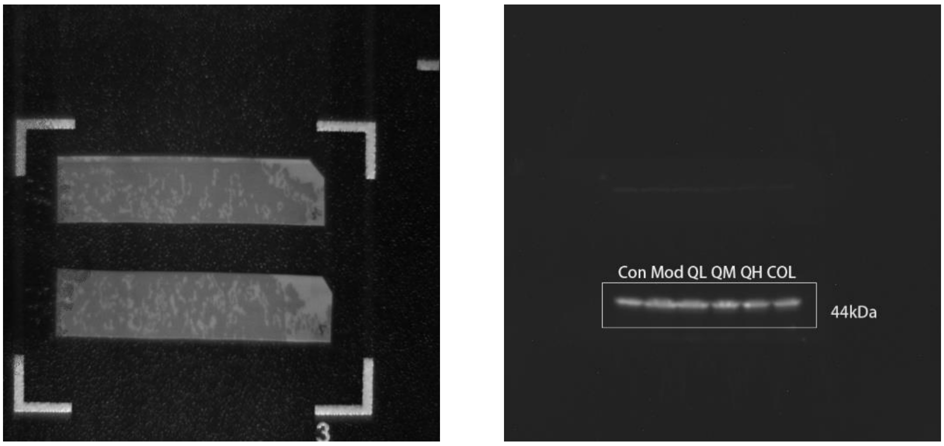

Original Image for Fig3-D-TGF-β1

p-Smad2

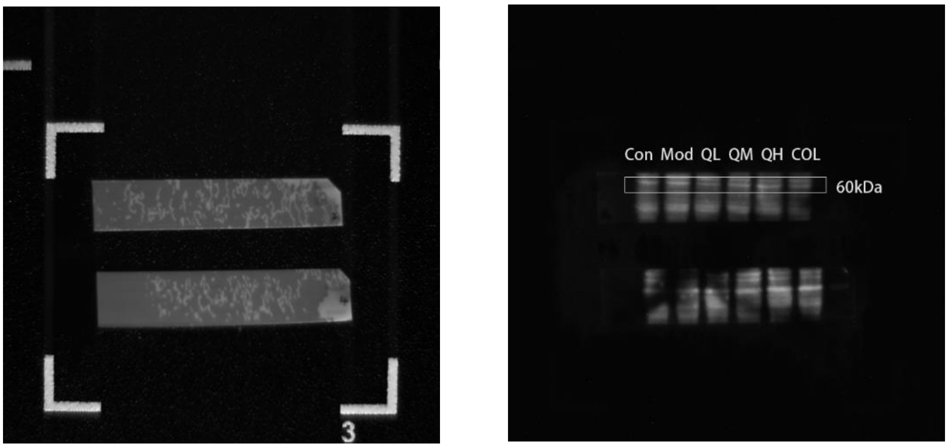

Original Image for Fig3-D-p-Smad2

Smad2

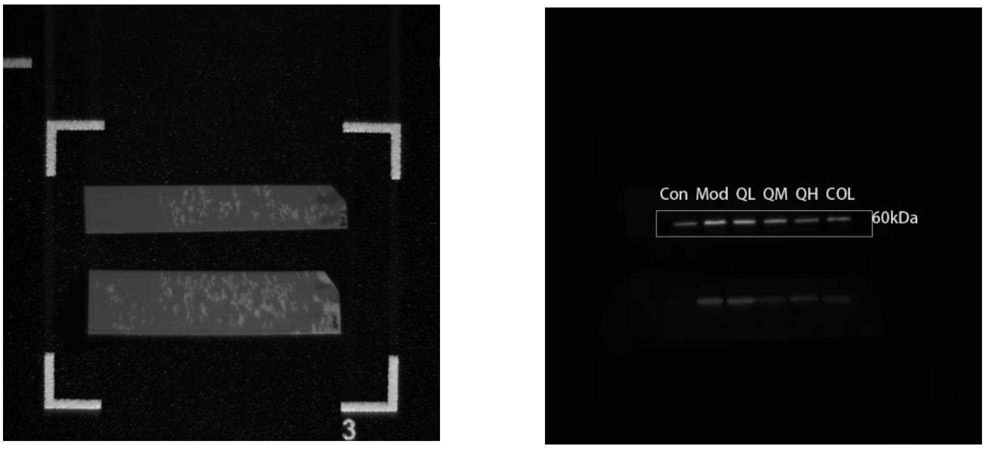

Original Image for Fig3-D-Smad2

p-Smad3

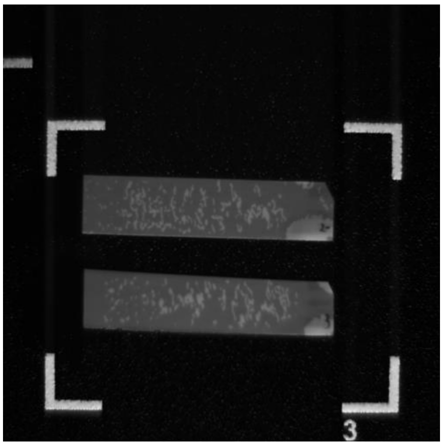

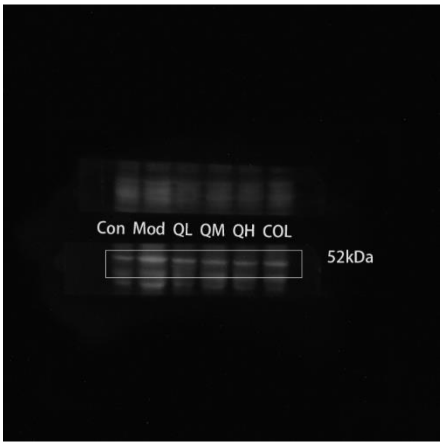

Original Image for Fig3-D-p-Smad3

Smad3

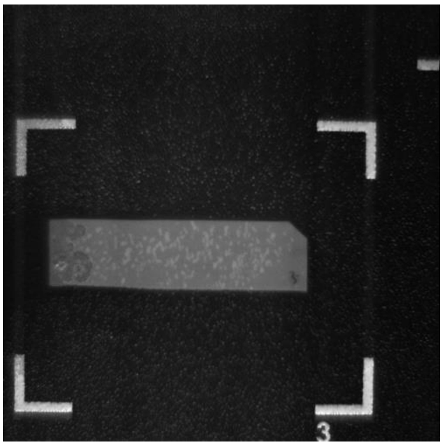

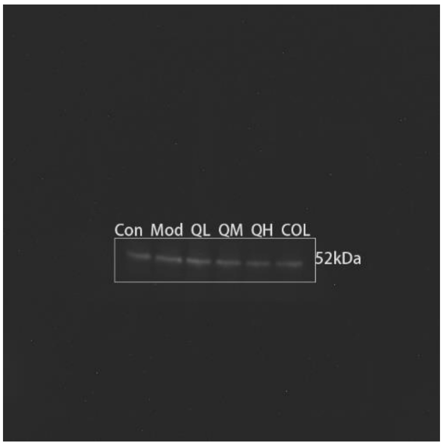

Original Image for Fig3-D-Smad3

α-SMA

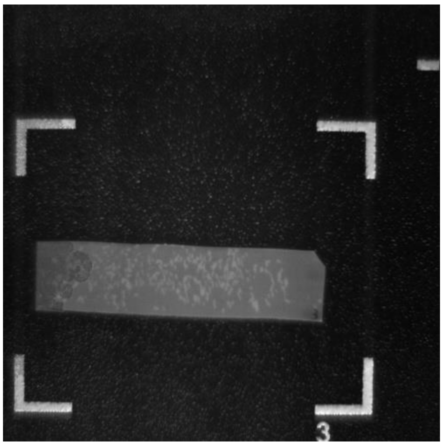

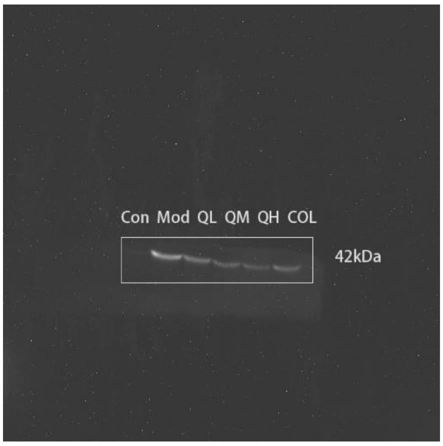

Original Image for Fig3-D-α-SMA

β-actin

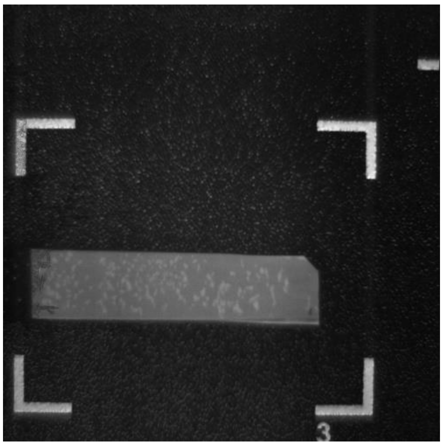

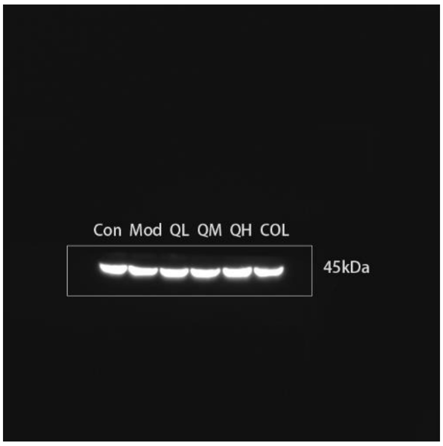

Original Image for Fig3-D-β-actin

α-SMA

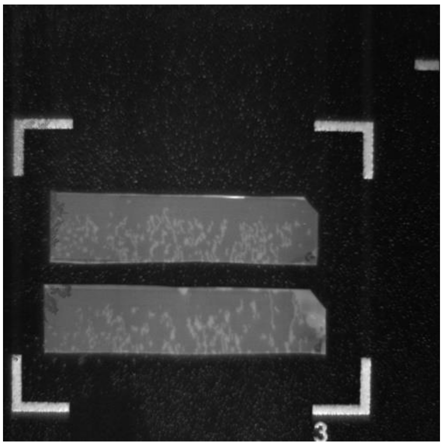

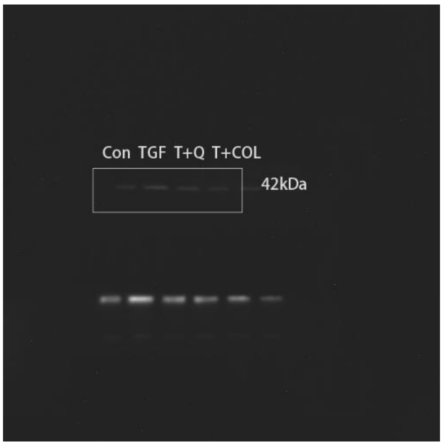

Original Image for Fig4-D-α-SMA

COL-1

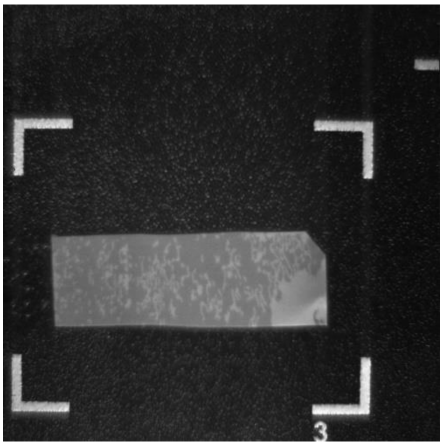

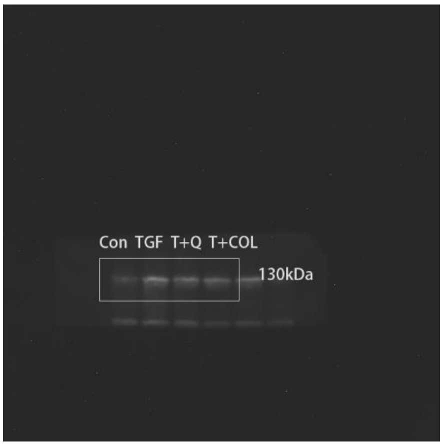

Original Image for Fig4-D-COL-1

β-actin

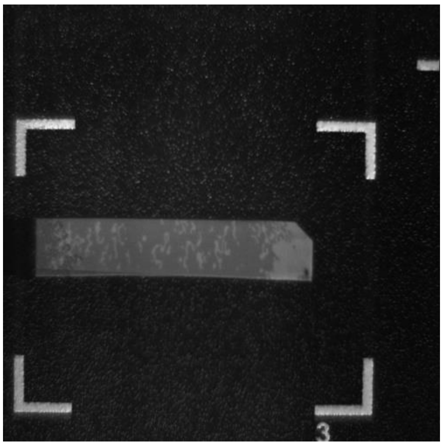

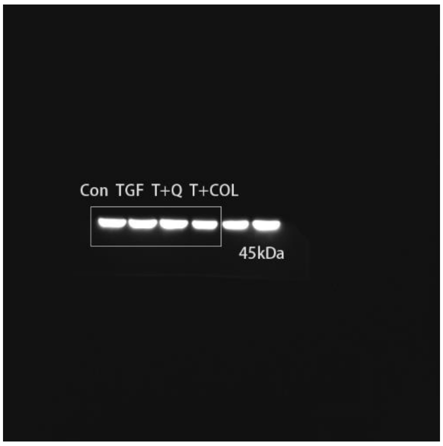

Original Image for Fig4-D-β-actin

TGF-βR1

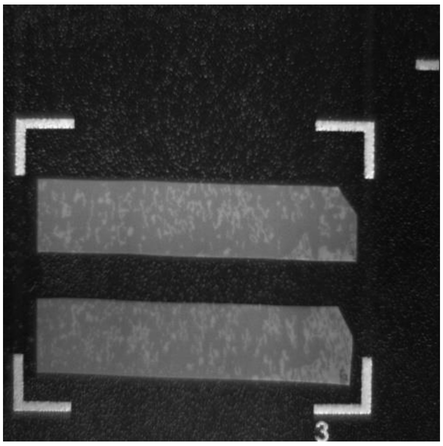

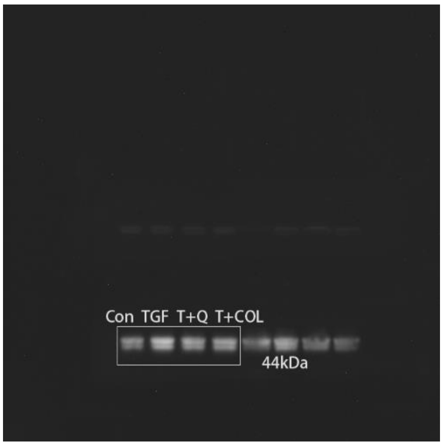

Original Image for Fig5-A-TGF-βR1

p-Smad2

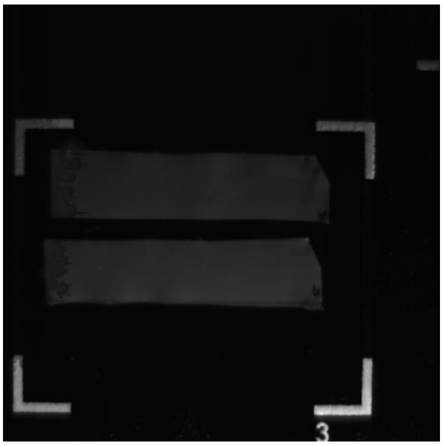

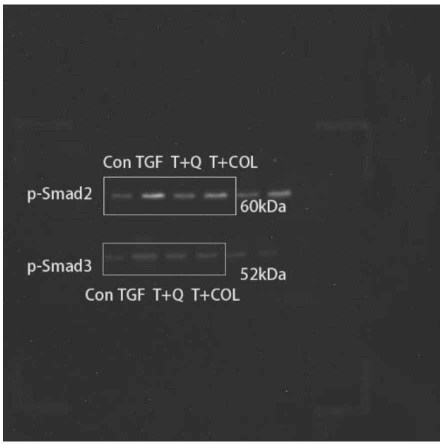

Original Image for Fig5-A-p-Smad2

Smad2

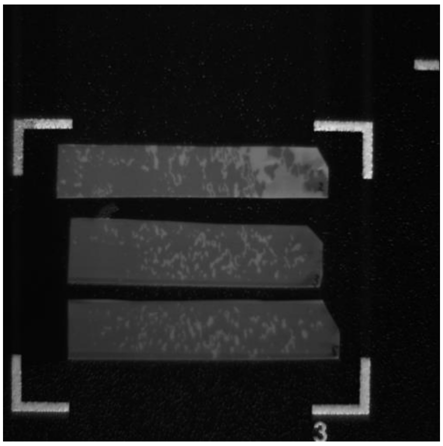

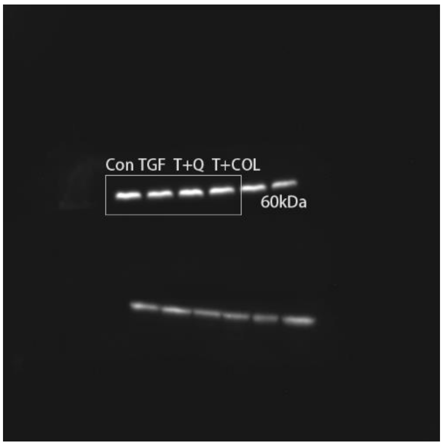

Original Image for Fig5-A-Smad2

p-Smad3

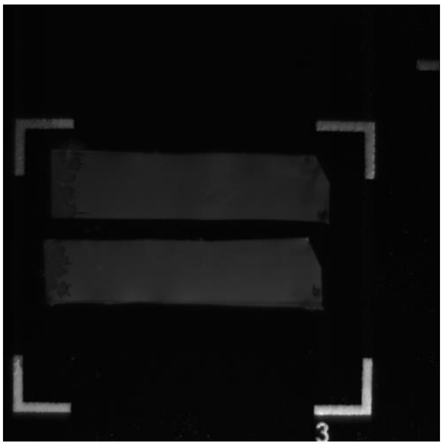

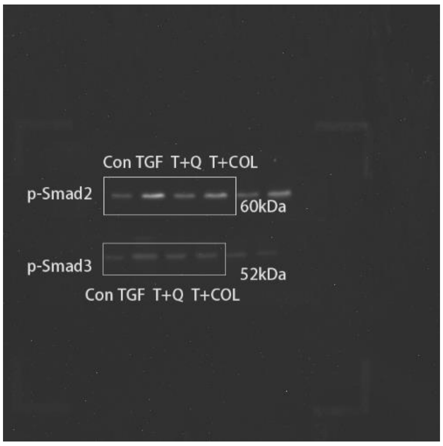

Original Image for Fig5-A-p-Smad3

Smad3

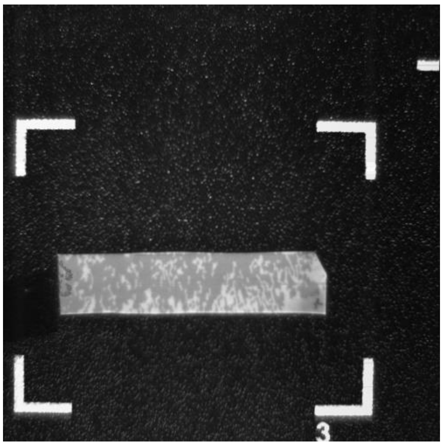

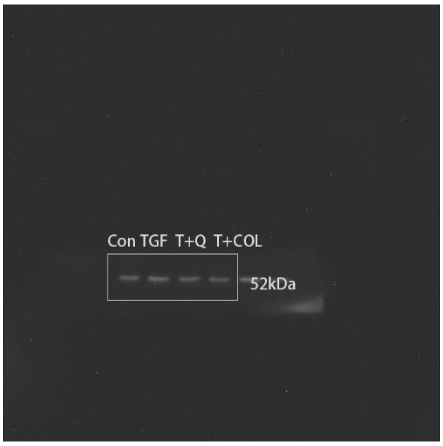

Original Image for Fig5-A-Smad3

β-actin

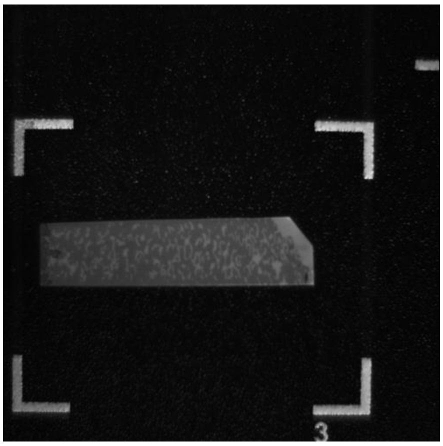

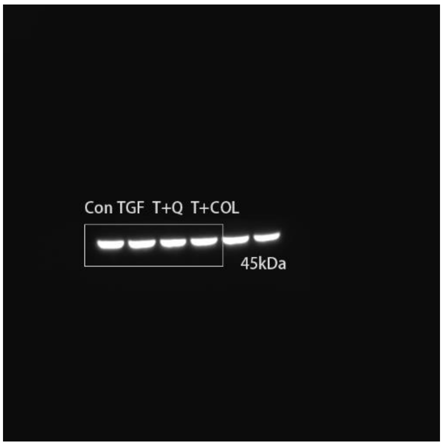

Original Image for Fig5-A-β-actin
