# Supplementary material for: Qinggan Yipi capsule ameliorates hepatic fibrosis in rats by down-regulating the TGF-β1/Smad2/3 signaling pathway and improving gut microbiota imbalance
Source: Front Pharmacol. 2025 Jan 24;16:1525914. doi: 10.3389/fphar.2025.1525914 (PMC11802500; doi:10.3389/fphar.2025.1525914)
Supplement: Supplementary file 2 [file Table2.docx]

Supplementary Table 2 Drug active components and targets of QgYp

| Medication | Number | Chemical compound | Target spot |
| --- | --- | --- | --- |
| *Misgurnus anguillicaudatus* | 1 | Succinic acid | EGLN1 |
|  | 2 | Butanoic acid | HDAC3 |
|  | 3 | Cadaverine | CA7、CA3、CA6、CA12、CA14、CA9、CA4、CA5B、CA5A、CA13 |
|  | 4 | Spermidine | CA2、CA7、CA1、CA6、CA14、CA9、CA4、CA5B、CA5A、CASP2 |
|  | 5 | Glutamic acid | GRM5、GRM2、GRIK1、GRIA1、ADORA3、GRIK5、SLC1A1、GRM4、GRM3、GRIA4、GRM8、GRIK2、GRIK3、GRM1、GRM7、GRIA2、GRM6、SLC1A2 |
|  | 6 | Nicotinic acid | HCAR2 |
|  | 7 | Xanthine | ACHE、GDA |
|  | 8 | Guanylic acid | HPRT1 |
|  | 9 | Deoxyguanylic acid | HPRT1 |
|  | 10 | Vitamin A | RBP4 |
|  | 11 | Adenylic acid | SRC、FBP1 |
|  | 12 | Inosinic acid | HPRT1 |
| *Bombyx batryticatus* | 1 | Palmitic acid | FABP4、PPARA、FABP3、FABP5、PPARD、FABP2、FFAR1、SLC22A6 |
|  | 2 | Palmitamide | CA2、CA1、PAOX |
|  | 3 | Beauverolide H | CETP、SOAT1、GHSR |
| *Indigo naturali*s | 1 | Beta-sitosterol | HMGCR、CYP51A1、AR、NPC1L1、NR1H3、CYP17A1、RORC、CYP19A1、ESR2、ESR1、PGR、NCOA2、PTGS1、PIK3CG、KCNH2、CHRM3、CHRM1、SCN5A、GABRA2、CHRM4、HTR2A、GABRA5、ADRA1A、GABRA3、CHRM2、ADRA1B、ADRB2、CHRNA2、SLC6A4、OPRM1、GABRA1、CHRNA7、BCL2、BAX、CASP9、CASP3、CASP8、PRKCA、TGFB1、PON1、MAP2 |
|  | 2 | Isovitexin | AKR1B1、PTGS2、AR、RELA、IKBKB、TNF |
|  | 3 | Indican | NOS2、MAOB |
|  | 4 | Indigo | RXRA、CCNA2 |
|  | 5 | (6E)-6-(3-oxo-1H-indol-2-ylidene)indolo[2,1-b]quinazolin-12-one | ESR1、KDR |
|  | 6 | Indirubin | FLT3、CDK5R1、CDK5、CCNB3、CDK1、CCNB1、CCNB2、MAPK14、GSK3B、CHEK1、PPARG、CA2、ACHE、CYP1A1、IFNG、AHR、CCL5、NQO2、CDKN3 |
| *Common to Misgurnus anguillicaudatus and Bombyx batryticatus* | 1 | Palmitoleic acid 9-hexadecenoic acid" | PPARG、PPARA、PPARD、FABP4、FABP3、FABP5、FAAH、TERT、FABP1、SCD、FFAR1 |
