# Supplementary material for: Qinggan Yipi capsule ameliorates hepatic fibrosis in rats by down-regulating the TGF-β1/Smad2/3 signaling pathway and improving gut microbiota imbalance
Source: Front Pharmacol. 2025 Jan 24;16:1525914. doi: 10.3389/fphar.2025.1525914 (PMC11802500; doi:10.3389/fphar.2025.1525914)
Supplement: Supplementary file 4 [file DataSheet1.docx]

**1.Experimental Materials**

Serum lipopolysaccharide (LPS), tissue lipopolysaccharide ELISA kit (Nanjing Jiancheng Bioengineering Institute, Batch No.: 20230913, Batch No.: 20231016).

**2. Enzyme-linked immunosorbent test (ELISA)**

According to the instructions of the ELISA assay kit, detect the levels of LPS in the colon, portal vein serum, and liver of rats.

1. **Effects of QgYp on LPS Levels in the Colon Tissue, Portal Vein Serum, and Liver Tissue of Rats with Liver Fibrosis**

As shown in the Supplementary Figure 1, compared to the Con group, the levels of LPS in the colon, portal vein serum, and liver tissue in the Mod group were significantly increased; after administration of QgYp, the levels of all three were significantly decreased. These results indicate that QgYp can inhibit the production of LPS.


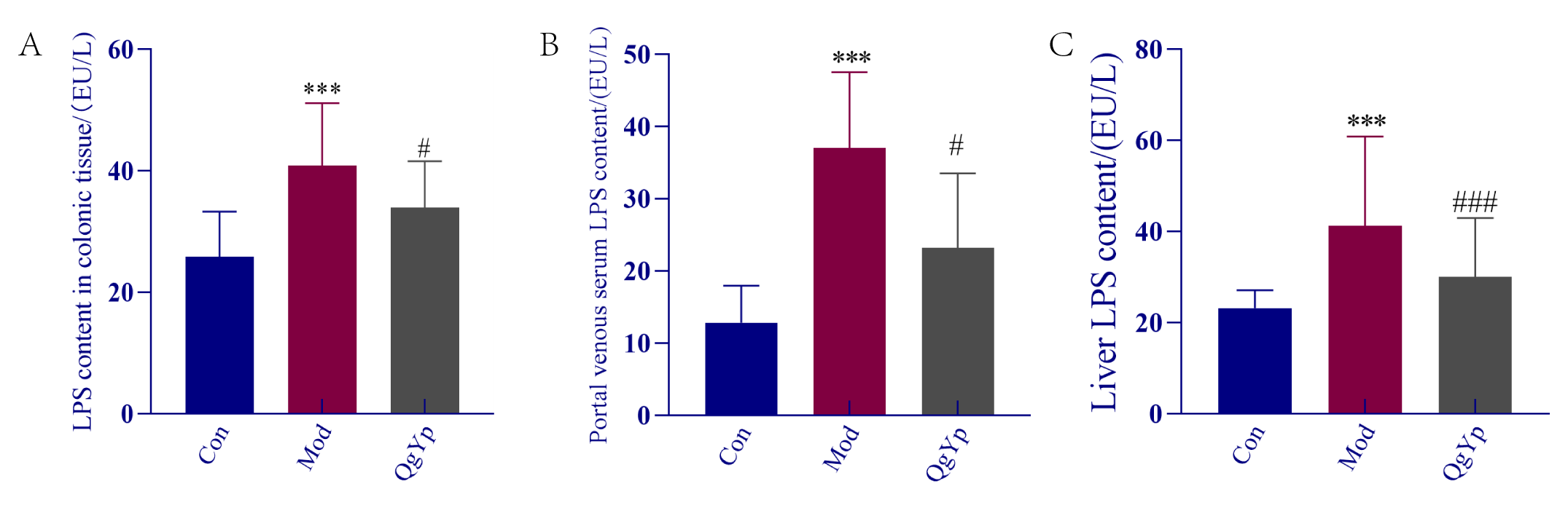


**Supplementary Figure 1 QgYp suppresses the production of LPS.** LPS levels in colonic tissue **(A)**, portal vein serum **(B)** and liver tissue **(C)** in liver fibrosis（±s，n=5）. ****P*<0.001 vs control group; ^#^*P*<0.05, ^###^*P*<0.001vs model group.
